# Design, Synthesis, and Evaluation of Novel Thiazole-Containing Algicides Inspired by Bacillamide A

**DOI:** 10.3390/md22110494

**Published:** 2024-11-01

**Authors:** Xiaoxue Li, Huili Li, Lei Shi, Zuguang Yin, Yuguo Du, Hongxia Zhang, Xin Wang, Xinxin Wang, Kexin Xu, Weili Wang, Ronglian Xing, Yi Liu

**Affiliations:** 1 School of Chemistry and Chemical Engineering, Yantai University, Yantai 264005, China; lixiaoxue0829@163.com (X.L.); sl17865561083@163.com (L.S.); y18354896509@outlook.com (Z.Y.); 2 School of Life Sciences, Yantai University, Yantai 264005, China; 13256952529@163.com (H.L.); zhxia128@163.com (H.Z.); j977158314@126.com (X.W.); twist991117@163.com (X.W.); 13256493559@163.com (K.X.); 3 State Key Laboratory of Environmental Chemistry and Eco-Toxicology, Research Center for Eco-Environmental Sciences, Chinese Academy of Sciences, Beijing 100085, China; duyuguo@rcees.ac.cn; 4 School of Chemistry and Material Science, Ludong University, 186 Hongqi Middle Road, Yantai 264025, China; weili.wang@ldu.edu.cn

**Keywords:** algicidal activity, harmful algal blooms, Bacillamide A, thiazole-containing derivatives, allelopathic algicide, SAR

## Abstract

The pursuit of highly effective, low-toxicity, and eco-friendly algicides for controlling and eradicating harmful algal blooms (HABs) is of paramount importance. The natural allelochemical bacillamide A has displayed impressive algicidal activity against harmful algae with favorable safety profiles. However, the poor synthetic efficiency and large dose requirements of bacillamide A limit its further application. In this paper, 17 thiazole-containing bacillamide derivatives (BDs) were designed and synthesized in three linear steps as potential algicides. Eight compounds (**6a**, **6c**, **6j**, **7b**, **7c**, **7d**, **7e**, and **7g**) displayed potent inhibitory effects against *Prorocentrum minimum*, *Skeletonema costatum*, and *Alexandrium pacificum*, and they had similar or better activity than the positive control (CuSO_4_) and bacillamide A. Compound **6a** exhibited the most potent algicidal activity against *S. costatum* (half-maximal effective concentration [EC_50_] = 0.11 μg/mL), being 23-fold more potent than bacillamide A, 28-fold more potent than CuSO_4_, and 39-fold more potent than Diuron. Compound **6j** exhibited significant algicidal activity against the toxic dinoflagellates *P. minimum* (EC_50_ = 1.0 μg/mL) and *A. pacificum* (EC_50_ = 0.47 μg/mL), being 3–5-fold more potent than natural bacillamide A, Diuron, and CuSO_4_. Micrographs and SEM images revealed that **6j** induced cell wall rupture and cellular content leakage. Biochemical and physiological studies indicated that **6j** might partially disrupt the antioxidant and photosynthetic systems in algal cells, resulting in morphological changes, cell wall rupture, and inclusion leakage. Our work suggests that **6j** has a distinct mode of action from CuSO_4_ and provides a promising candidate for the development of new algicides, worthy of further investigation.

## 1. Introduction

Annual harmful algal blooms (HABs) have caused continuous health and environmental concerns over the last decade, as they are associated with numerous adverse effects such as seafood poisoning, economic losses, environmental damage, and pandemics [1]. Global climate change stressors (e.g., increasing temperatures, water acidification, ocean deoxygenation) and anthropogenic factors (e.g., discharge from wastewater treatment plants, aquaculture, marine transportation, ballast water transfer) have broadened the geographic distribution and frequency of HABs [2,3].

Furthermore, HABs produce a variety of potent naturally occurring biotoxins, including neurotoxic shellfish toxins, diarrhetic shellfish toxins, and paralytic shellfish toxins (PSTs) [4]. Unavoidably, these toxins can accumulate in the tissues of bivalve shellfish, fish, and other marine creatures. The consumption of poisoned seafood leads to severe and potentially fatal poisoning [5]. HAB toxins are responsible for 50,000–500,000 cases of intoxication annually, and they contribute to a global mortality rate of approximately 1.5% each year [6]. Marine toxigenic dinoflagellates such as *Gymnodinium catenatum*, *Alexandrium spp*., and *Pyrodinium bahamense var*. compressum are the major producers of PSTs [7]. In 2021, *A. pacificum* was identified as the major producer of PSTs in China [8].

To date, a number of biological, physical, and chemical control techniques have been investigated to control HABs [9]. Although some physical and chemical methods can efficiently control and remove HABs, they are limited by drawbacks such as high time consumption and costs, non-target toxicity, and secondary pollution from heavy metals. Biological algicidal methods have attracted significant attention as environmentally friendly alternatives [10]. However, most of these methods remain at the laboratory scale, and their effectiveness in managing natural HABs is unclear. Therefore, there is an urgent need for novel, environmentally friendly algicides to control and eradicate HABs.

**Figure 1:**
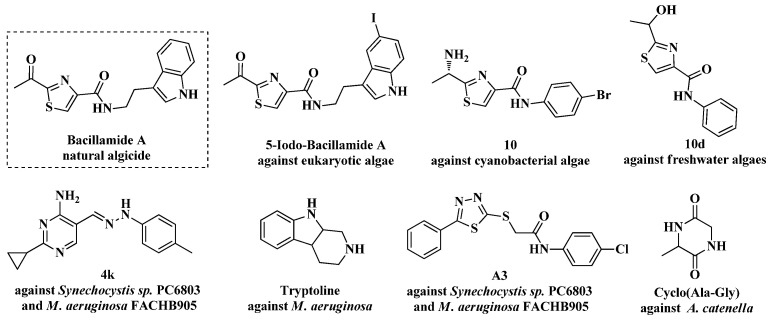
Bacillamide A and reported algicides.

Allelopathic algicides have attracted considerable attention because of their high selectivity, high sustainability, and negligible toxicity in controlling HABs [11]. In 2003, the thiazole-containing natural algicide bacillamide A was first isolated from marine bacterium *Bacillus* sp. SY-1 by Okada group [12]. Bacillamide A exhibited excellent and selective algicidal activity against harmful dinoflagellates, including *Cochlodinium polykrikoides* (50% lethal concentration [LC_50_] = 3.2 μg/mL), *Gyrodinium impudicum* (LC_50_ = 2.3 μg/mL), *Alexandrium catenella* (LC_50_ = 9.4 μg/mL), *Prorocentrum micans* (LC_50_ = 4.4 μg/mL), and the raphidophytes *Heterosigma akashiwo* (LC_50_ = 1.6 μg/mL) and *Chattonella* sp. (LC_50_ = 3.7 μg/mL). Comparatively, bacillamide A did not display algicidal activities against other microalgae (e.g., diatoms, cyanobacteria, green algae) [12,13]. Further studies revealed that bacillamide A exhibited moderate antibacterial activity against Gram-positive bacteria (*Salmonella choleraesuis* and *Vibrio cholerae*) and slight antifungal activity against *Alternaria* sp. [14]. The potent algicidal activity of bacillamide A has attracted attention from synthetic chemists and biologists. Recently, several promising algaecides have been reported, including pyrimidin-4-amine (**4k**) [15], 1,3,4-thiadiazole-containing (A3) algicides [16], cyclo(Ala-Gly) [17], natural allelopathic algicides [18], and several Bacillamide derivatives (BDs) [19,20] (Figure 1).

Because of its high algicidal potency and distinct structure characteristics and our continuing focus on the total synthesis of thiazole-containing natural products, bacillamide A has attracted our attention [21]. However, large-scale preparation and structural modifications of bacillamide A have remained difficult. Therefore, structurally simplified and detailed structural modifications of bacillamide A would provide an opportunity for the development of highly effective, environmentally friendly algicides.

## 2. Results and Discussion

### 2.1. Chemical Synthesis

**Scheme 1:**
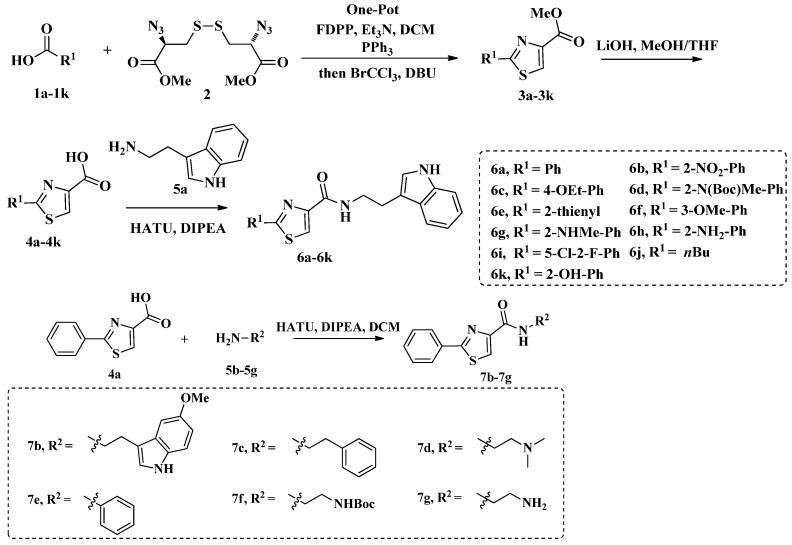
General synthesis routes of bacillamide derivatives (**6a**–**6k** and **7b**–**7g**).

The natural algicide bacillamide A was prepared according to our previously reported synthesis route [21]. The synthesis route for BDs is presented in Scheme 1. The synthetic route of **6a**–**6k** began with the formation of the 2, 4-disubstituted thiazoles **3a**–**3k** following our reported cascade thiazole formation reaction, with the yields reaching 63–75% [22]. After saponification of **3a**–**3k**, the acids **4a**–**4k** were next reacted with tryptamine, generating BDs **6a**–**6k** in 45–67% yields. To explore the structure–activity relationship of the tryptamine unit, the thiazole **4a** was coupled with different amines to generate **7b**–**7g** in 44–51% yields for two steps. The structures of all synthesized BDs were characterized by ^1^H and ^13^C NMR spectra and high-resolution mass spectrometry (HRMS).

### 2.2. Algicidal Activity Assay and Structure–Activity Relationship Discussion

**Figure 2:**
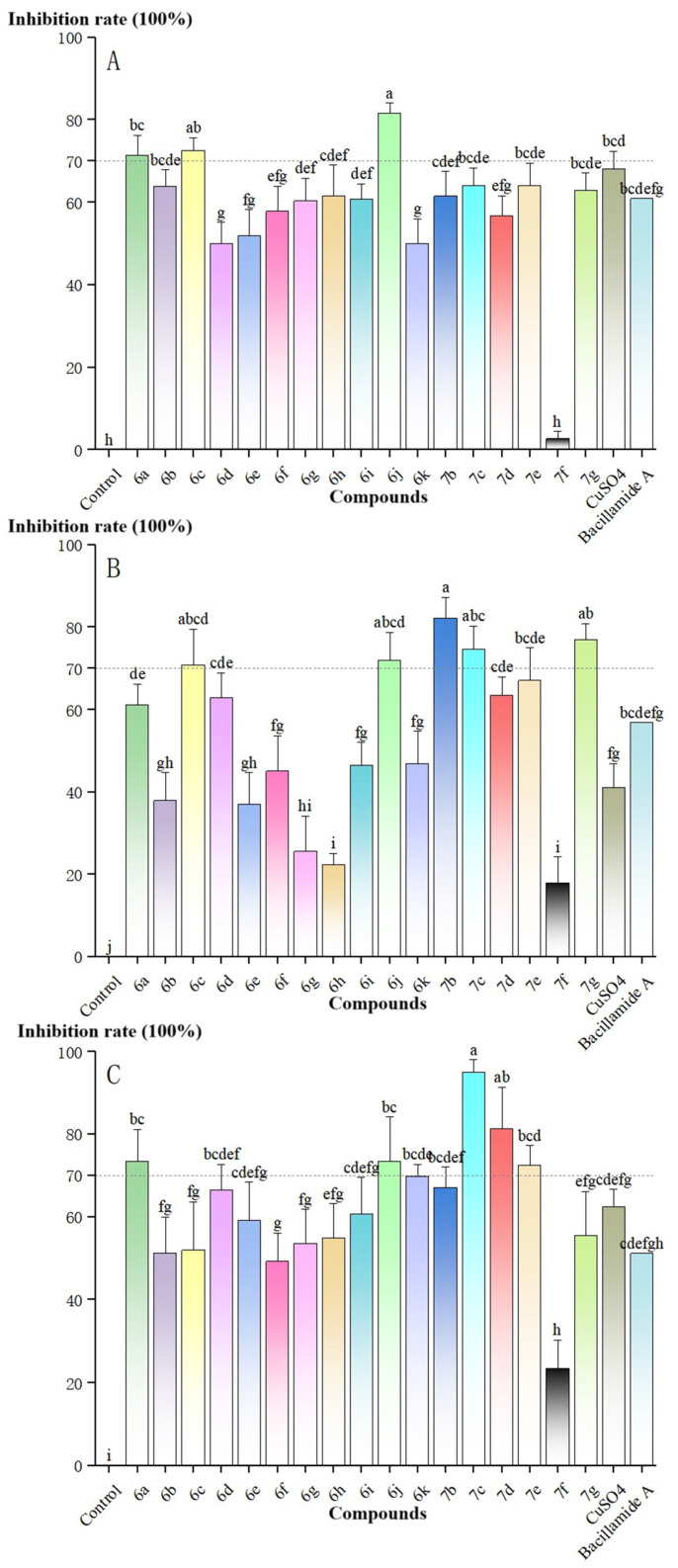
In vitro algicidal inhibition rates of synthesized BDs, bacillamide A, and CuSO_4_ against the growth of *S. costatum* (**A**), *P. minimum* (**B**), and *A. pacificum* (**C**) at 50 mg/L for 24 h. Mean ± SD, n = 6. Different letters represent significant differences between groups, a > b > c > d > e > f > g > h > i > j (*p* < 0.05), and the same letters indicate no significant differences between groups.

The inhibitory activities of bacillamide A and 17 BDs were preliminarily evaluated in three algae (*P. minimum, S. costatum, and A. pacificum*) to identify algicidal candidates. *S. costatum* is a single-celled diatom-type phytoplankton, whereas *P. minimum* and *A. pacificum* are toxic dinoflagellates. As presented in Figure 2, most of the synthesized BDs exhibited similar or better algicidal activity than bacillamide A and CuSO_4_ after co-incubation for 24 h at a concentration of 50 mg/L. Notably, **6a**, **6c**, and **6j** exhibited >70% inhibition rates against *S. costatum* at 50 mg/L. Replacing the acetyl group of bacillamide A with 4-OEt-Ph (**6c**) or butyl (**6j**) significantly improved their inhibition rates (**6c**: 72.6%; **6j**: 81.7%) against *S. costatum*. However, substitution of phenyl groups or incorporation of the heteocyclic thiophene ring had little effect on their algicidal activity. Excluding the large steric hindrance of the Boc group (**7f**), replacing the tryptamine group in bacillamide A with 5-methoxytryptamine (**7b**), phenylethyl (**7c**), phenyl (**7e**), aminoethyl (**7g**), or dimethylaminoethyl (**7d**) did not result in significant decreases in algicidal activity, indicating that the thiazole unit was more important for maintaining algicidal potency.

As depicted in Figure 2B,C, the BDs with a thiazole skeleton also exhibited significant algicidal inhibition rates against the toxic dinoflagellates. Five thiazole-containing BDs all displayed inhibition rates exceeding 70% (**6c**: 70.8%; **6j**: 71.9%; **7b**: 82.3%; **7c**: 74.7%; and **7g**: 76.9%) against the growth of *P. minimum*. Compounds **6a**, **6j**, **7c**, **7d**, and **7e** substantially inhibited *A. pacificum* growth with inhibition rates of 70–95%, exceeding those of bacillamide A (55.3%) and CuSO_4_ (62.3%). After treatment for 24 h, **7c** displayed the most powerful inhibitory activity against *A. pacificum* at 50 mg/L. By contrast, **7f** lost most of its algicidal activity against *P. minimum* and *A. pacificum*. Notably, the inhibition rates of **6j** exceeded 70% for all three algal species. Regarding the suppression of dinoflagellates, the selectivity of **7b**–**7g** progressively increased, demonstrating that R^2^ substitution resulted in better inhibition of dinoflagellate growth than did substitution at R^1^.

To further evaluate algicidal efficacy of the synthesized BDs, those with inhibition rates exceeding 70% at 50 mg/L were selected for half-maximal effective concentration (EC_50_) testing. As presented in Table 1, most of the synthesized BDs exhibited better algicidal activity than the commercial positive controls (CuSO_4_ and Diuron). The BDs with the same tryptamine unit were analyzed, and the sequence of algicidal activity against *S. costatum* was **6a** (phenyl) > **6j** (butyl) > **6c** (4-OEt-phenyl). Compound **6a** exhibited the best algicidal activity against *S. costatum* (EC_50_ = 0.11 μg/mL), being 28-fold more potent than CuSO_4_, 39-fold more potent than Diuron, and 23-fold more potent than bacillamide A. However, compared with **6a** and **6c**, **6j** exhibited better algicidal activity against *P. minimum* (EC_50_ = 1.0 μg/mL) and *A. pacificum* (EC_50_ = 0.47 μg/mL). Five synthesized phenylthiazole-containing BDs (**7b**, **7c**, **7d**, **7e**, and **7g**) exhibited good algicidal activity against *S. costatum* (EC_50_ = 0.24–2.28 μg/mL), *P. minimum* (EC_50_ = 1.32–3.38 μg/mL), and *A. pacificum* (EC_50_ = 0.53–3.42 μg/mL), demonstrating that the phenylthiazole unit was important for algicidal activity maintenance.

### 2.3. Acute Toxicity Evaluation of BDs

To explore the safety profile of the synthesized BDs, the 2-phenylthiazole- and 2-butylthiazole-containing BDs (**6a** and **6j**) with the best algicidal activity were selected as representative compounds for further fish toxicity evaluation (Table S1). The Guide for Environmental Safety Assessment of Chemical Pesticides (GB/T31270.12) has documented the standards for toxicity (LC_50 (96 h)_ > 10 mg/L, low toxicity; 1.0 < LC_50 (96 h)_ ≤ 10 mg/L, moderate toxicity; LC_50 (96 h)_ ≤ 0.1 mg/L, high toxicity). As seen in Table S1, after treatment with bacillamide A at 0.1 mg/L, 1 mg/L, and 10 mg/L for 0 h, 24 h, 48 h, 72 h, and 96 h, the zebrafish survival rates were 100%. Therefore, the acute toxicity of bacillamide A was classified as low. CuSO_4_ at 10 mg/L all led to 100% zebrafish mortality within 48 h. Unfortunately, the acute toxicity of both **6a** and **6j** was classified as moderate. **6j** exhibited a better safety profile than **6a**. Although the survival rate of zebrafish after 24 h of treatment with **6j** at 10 mg/L was up to 80%, treatment with **6j** for more than 48 h also led to 100% mortality in zebrafish. These results suggest that bacillamide A has excellent biosafety in aquatic animals, and alkyl modification of the thiazole ring could represent a strategy for safe algicide development in the future.

### 2.4. Effects of BDs on the Cellular Morphology of P. minimum, S. costatum, and A. pacificum

**Figure 3:**
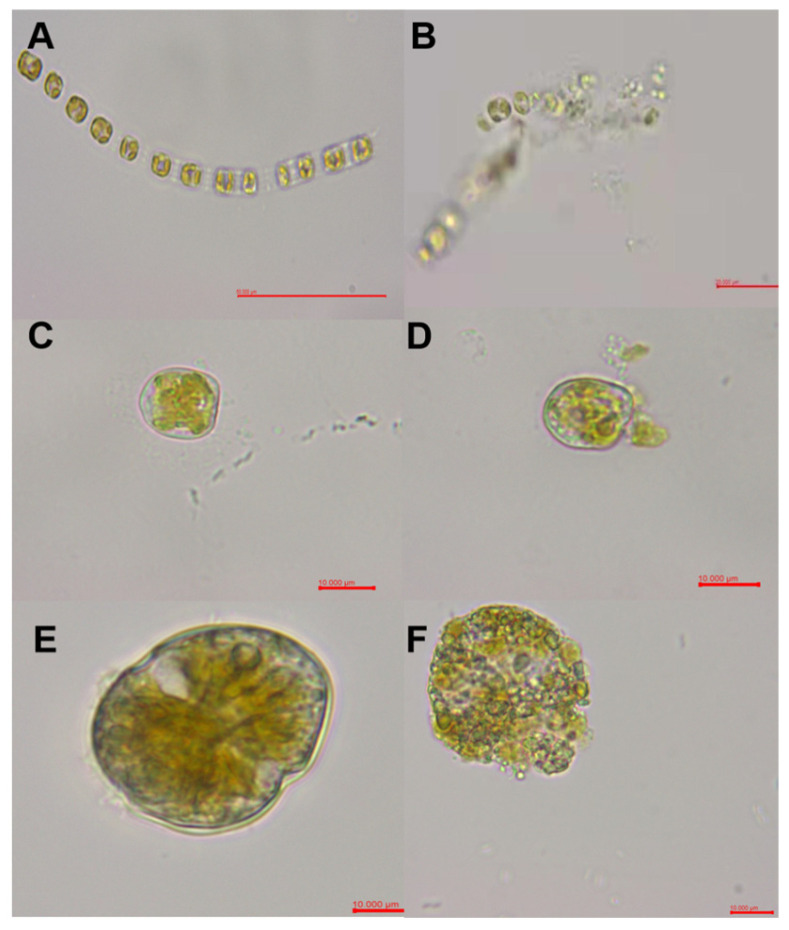
The effects of **6j** on the cell morphology of *S. costatum*, *P. minimum*, and *A. pacificum*. The algae cells were incubated with **6j** at the concentration of 10 mg/L for 4 h. The micrographs showing the effects of *S. costatum* (**A**), *P. minimum* (**C**), and *A. pacificum* (**E**) without treatment with **6j**. The micrographs showing the effects of **6j** on *S. costatum* (**B**), *P. minimum* (**D**), and *A. pacificum* (**F**).

Based on the excellent algicidal characteristics of **6j**, the effect on the cellular morphology of algae was next evaluated. As presented in Figure 3A,C,E, the three untreated algae (*S. costatum*, *P. minimum*, and *A. pacificum*) had well-defined shapes and smooth surfaces. After treatment with **6j** (10 mg/L), the micrographs illustrated that the cell walls of the treated algae (*S. costatum*, *P. minimum*, and *A. pacificum*) were broken or damaged or both and that there was leakage of the cytoplasm (Figure 3B,D,F).

**Figure 4:**
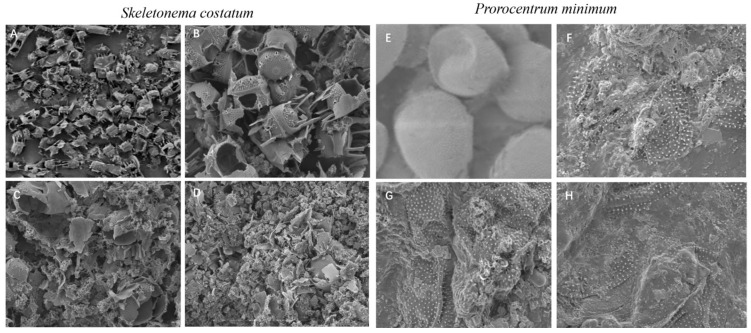
SEM images presenting the effects of treatment with DMSO (**A**), bacillamide A (**B**), **6j** (**C**), and CuSO_4_ (**D**) on the cellular morphology of *S. costatum*. SEM images presenting the effects of treatment with DMSO (**E**), bacillamide A (**F**), **6j** (**G**), and CuSO_4_ (**H**) on the cellular morphology of *P. minimum*. Scale bar = 10 μM.

Scanning electron microscope photographs showed that the untreated *S. costatum* and *P. minimum* also exhibited smooth surfaces, and the cells were arranged in orderly strings (Figure 4A,E). After incubation with **6j** (10 mg/L) for 4 h, **6j**-treated algal cells of *S. costatum* and *P. minimum* had rough and broken surfaces, and the disappearance of cytoplasm and nucleoid regions was observed (Figure 4C,G). The *P. minimum* cells had almost completely lysed, and they lost their integrity. Bacillamide A exhibited similar effects to that of **6j** on the morphology of algae cells, but the algae cell walls were broken down more severely by copper sulfate under SEM observation (Figure 4B,F,D,H). These results demonstrate that **6j** can cause significant cell wall damage and change the subcellular structure and morphology of *S. costatum* and *P. minimum* cells, highlighting its strong algicidal activity.

### 2.5. Preliminary Exploration of the Algicidal Action of **6j**

In algal cells, photosystem II (PSII) was a crucial supramolecular complex located within thylakoid membranes. PSII is responsible for harvesting light energy, stimulating charge separation, and driving the electron transport process. These biological processes can effectively split the H_2_O into protons and oxygen in algal cell under ambient temperature [23]. PSII dysfunction can lead to the failure to harvest light energy and lower the electron transfer rate across thylakoid membranes to photosystem I (PSI), resulting in the accumulation of reactive oxygen species (ROS).

**Figure 5:**
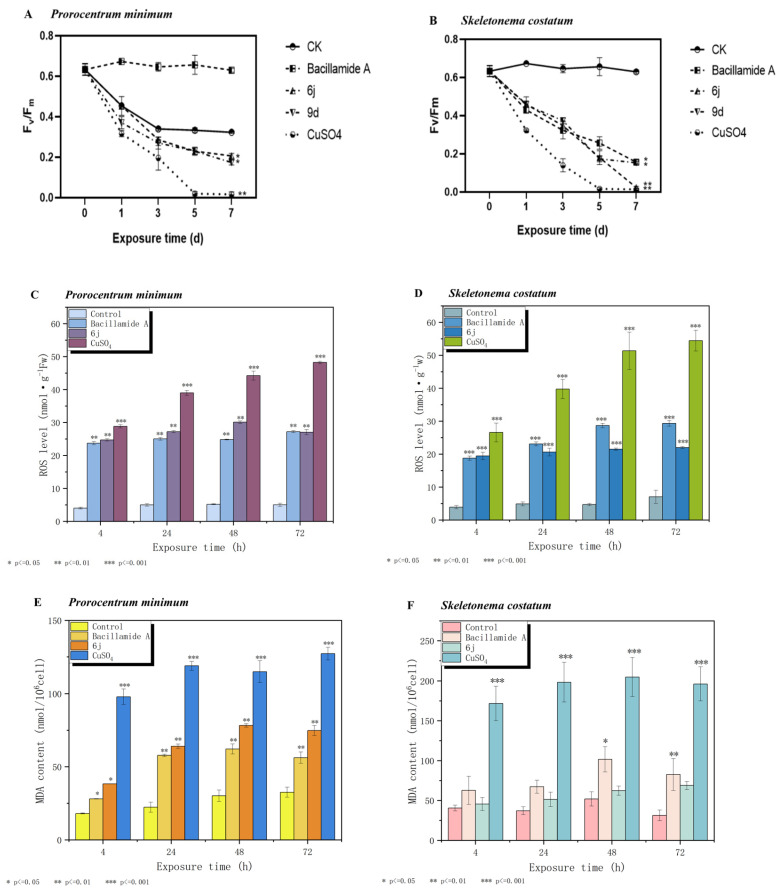
The effect of **6j** on the Fv/Fm of PSII in *P. minimum* (**A**) and *S. costatum* (**B**). (**C**) Effects of bacillamide A, DMSO, CuSO_4_, and **6j** on ROS content levels in *P. minimum*. (**D**) Effects of bacillamide A, DMSO, CuSO_4_, and **6j** on ROS content levels in *S. costatum*. (**E**) Effects of bacillamide A, DMSO, CuSO_4_, and **6j** on MDA content levels in *P. minimum*. (**F**) Effects of bacillamide A, DMSO, CuSO_4_, and **6j** on MDA content levels in *S. costatum*. DMSO was used as the negative control. CuSO_4_ was used as the positive control. Mean ± SD, *n* = 3. * *p* < 0.05, ** *p* < 0.01, *** *p* < 0.001.

As illustrated in Figure 5A,B, compared with the effects of the negative control, the quantum yield Fv/Fm of PSII in *P. minimum* and *S. costatum* decreased after treatment with 10 mg/L of **6j**, bacillamide A, and CuSO_4_. After 72 h of exposure to bacillamide A or **6j**, the quantum yield Fv/Fm of PSIIin *S. costatum* decreased by 4- and 27-fold, respectively. Meanwhile, treatment with CuSO_4_ led to a 48-fold reduction in the Fv/Fm. A similar tendency was observed in *P. minimum*, as treatment with bacillamide A and **6j** resulted in 2- and 3-fold decreases in the Fv/Fm of PSII after 72 h, respectively. However, CuSO_4_ treatment caused a 20-fold decrease in the Fv/Fm. These results demonstrated that **6j** could reduce the efficiency of PSII, suggesting that the algicidal mechanism of BDs differs from that of CuSO_4_.

Consistent with the reduction in the Fv/Fm, the ROS levels in *P. minimum* and *S. costatum* increased by 5.4- and 3-fold, respectively, after treatment with 10 mg/L of **6j** for 72 h (Figure 5C,D). A similar effect was observed after treatment with 10 mg/L of bacillamide A for 72 h. However, after treatment with 10 mg/L of CuSO_4_ for 72 h, the ROS levels in *P. minimum* and *S. costatum* significantly increased by 9.6- and 7.7-fold, respectively. These results indicate that both **6j** and bacillamide A, similarly to CuSO_4_, could increase ROS levels, leading to algal cell apoptosis.

Both microscopy and SEM revealed that treatment with **6j** lysed algal cells and destroyed their cell walls, prompting further exploration of its algicidal mechanism. Malondialdehyde (MDA) is the typical marker of lipid peroxidation [24]. As presented in Figure 5E,F, incubation with 10 mg/L of **6j** for 72 h increased MDA levels in *P. minimum* and *S. costatum* by 2.3- and 2.2-fold, respectively, versus the effects of the control (DMSO). Notably, bacillamide A treatment increased MDA levels by 2.0- and 2.6-fold, respectively, in these algal species. These findings suggest that the algicidal mode of action for both **6j** and bacillamide A involves reductions in the efficiency of PSII and increases in intracellular MDA and ROS levels, consequently causing algal cell rupture.

## 3. Materials and Methods

### 3.1. Instruments and Chemicals

Diuron (98% pure) and cupric sulfate anhydrous (98% pure) were supplied by China ACON Bitotech Co., Ltd. (Hangzhou, China). Tryptamine (98% pure) was supplied by China Heowns Biochem Technologies, LLC (Tianjin, China). Other chemicals (reagent grade) were purchased from commercial sources (InnoChem, Beijing, China). Flash chromatography (FC) silica gel (200–300 meshes, Qingdao Haiyang Chemical Go., Ltd., Qingdao, China) was used as the separating compound. The high-resolution mass spectra (HRMS), ^1^H NMR, and ^13^C NMR spectra (NMR in DMSO-*d*_6_, CDCl_3_ and CD_3_OD with TMS as internal standard) were tested by using a Q-TOF analyzer (Thermo Fisher Scientific, BRE, German), JEOL JNM-ECZ400S (400 MHz/100 MHz, Akishima, Japan) spectrometers, and Bruker spectrometers (500 and 125 MHz, Billerica, MA, USA), respectively. ^1^H NMR and ^13^C NMR spectra of BDs **6a**–**6k**, **7b**–**7g** were shown in Supplementary Materials. Compound **2** was obtained following our previous reported method [22]. Bacillamides A were prepared in 6 linear steps with 27% overall yield following our previous reported synthetic procedure [21].

### 3.2. Algal Species

*P. minimum*, *A. pacificum*, and *S. costatum* were purchased from Guangyu Biological Technology Co., Ltd. (Shanghai, China). The algae strain was cultured in F/2 medium ((12 h light: 12 h dark cycle), 20 ± 1 °C, 70 μmol m^−2^ s^−1^). Purchased algae were transferred to sterile conical flasks for culture; after the algae proliferation was stabilized, the expanded culture was next carried out following standard conditions.

### 3.3. Synthesis Procedure of BDs

#### 3.3.1. General Procedure for the Preparation of Thiazole Methyl Ester **3a**–**3k** [22,25]

A mixture of carboxylic acid **1** (6 mmol), pentafluorophenyl diphenylphosphinate (FDPP) (6 mmol), and triethylamine (TEA) (12 mmol) in 100 mL of CH_2_Cl_2_ were stirred at room temperature for 20 min. β-azido disulfide **2** (1.5 mmol) and PPh_3_ (12 mmol) was subsequently added into the mixture and heated at 45 °C for 5 h. After cooling to 0 °C, bromotrichloromethane (12 mmol) and 1,8-diazabicycloundec-7-ene (DBU) (16 mmol) were added into the solution via syringe over 20 min. The mixture was stirred for 1 h until reaction was complete. Reaction was quenched by 100 mL of saturated NH_4_Cl solution, and the mixture was extracted with DCM (200 mL × 3). The combined organic layer was washed with brine and dried over anhydrous Na_2_SO_4_, filtered, concentrated in vacuo (below 45 °C), and purified by flash chromatography to produce compound **3a**–**3k**.

#### 3.3.2. General Procedure for Hydrolysis of **3a**–**3k**

To a solution of LiOH (200 mg, 8.4 mmol) in H_2_O (20 mL)/MeOH (50 mL)/THF (50 mL) was added thiazole methyl ester **3a**–**3k** (10 mmol), and the reaction mixture was stirred at room temperature for 3–5 h. After the addition of 20 mL of H_2_O, the solution was acidified (pH 3–5) with 10% aqueous solution of 1.0 N HCl. The solution was diluted with EtOAc (50 mL × 3). The extracts were washed with saturated brine, dried (Na_2_SO_4_) and concentrated under reduced pressure to produce carboxylic acid **4a**–**4k**.

#### 3.3.3. General Procedure the Preparation of BDs **6a**–**6k** and **7b**–**7g**

A solution of acid **3a**–**3k** (1.0 mmol), DIPEA (2.0 mmol), and HATU (1.5 mmol) in 20 mL of CH_2_Cl_2_ was stirred at room temperature for 20 min. Amine **5a**–**5g** (1.0 mmol) was then added into the reaction mixture at 0 °C and stirred overnight. The solution was quenched by saturated aqueous NH_4_Cl and extracted with 30 mL of CH_2_Cl_2_ for 3 replicates. After that, the combined organic phase was washed with brine and dried (Na_2_SO_4_), and the solvent was removed by rotary evaporation. Crude residue was purified by flash chromatography to produce BDs **6a**–**6k** and **7b**–**7g**.

### 3.4. Algicidal Activity Assay

The stock solutions of bacillamide A and different BDs were prepared in dimethyl sulfoxide (DMSO) with the requisition amount. The initial cell density of three algae (*P. minimum, S. costatum, and A. pacificum*) was up to 1.0 × 10^6^ cells/L. A total of 20 mL of algae solution was exposed to bacillamide A and BDs at concentrations of 50 mg/L for growth-inhibition rate measurement (24 h). The controls received the same volume of DMSO. The inhibitory activities of bacillamide A, different BDs, and positive controls were screened by 96 well microtiter plates at different concentrations. Inhibition ratios (IRs) of cupric sulfate (50 mg/L, positive control), bacillamide A (50 mg/L), and BDs (50 mg/L) were calculated on algae using Equation (1).

(1) $$\mathrm{IR}=\frac{C_{c0}-C_{\mathrm{ex}}}{C_{c0}}\times 100\%$$

The C_C0_ and C_ex_, respectively, represent the algal densities of the control and experimental groups.

The half-maximal effective concentration (EC_50_) against three algae species were evaluated: 20 mL of algae solution was, respectively, exposed to Diuron (positive control), cupric sulfate (positive control), and BDs (concentration: 0.1 mg/L, 0.2 mg/L, 1 mg/L, 5 mg/L, and 10 mg/L, time: 0 d, 1 d, 3 d, 5 d, 7 d, 9 d, 11 d, and 13 d). Copper sulfate and Diuron were used as the positive controls. EC_50_ values were calculated according to the equation of the quantity–effect relationship in Equation (2) using the Hill model of Graph Pad Prism software (8.0.2).

(2) $$Y=\frac{\mathrm{Bottom}+(\mathrm{Top}-\mathrm{Bottom})}{{1+10}^{{(\mathrm{Log}\;\mathrm{EC}}_{50}-X)\times \mathrm{Hill}\;\mathrm{Slope}}}$$

The X and Y values represent the concentration (mg/L) and response values of the control and experimental groups, respectively. The Top, Bottom, and Hill Slope represent the maximum response, minimum response, and the curve slope, respectively. The EC50 represents half of the maximum effective concentration.

### 3.5. Zebrafish Acute Toxicity Assay of Bacillamide A and BDs

The Guide for Environmental Safety Assessment of Chemical Pesticides has documented the criteria for toxicity (GB/T31270.12, LC_50 (96 h)_ > 10 mg/L, low toxicity; 1.0 < LC_50 (96 h)_ ≤ 10 mg/L, moderate toxicity; LC_50 (96 h)_ ≤ 0.1 mg/L, high toxicity). Zebrafish acute toxicity of bacillamide A and synthesized BDs were evaluated at 0.1 mg/L, 1 mg/L, and 10 mg/L, respectively. Diuron and CuSO_4_ were used as positive controls. The commercial zebrafish (body length > 3.0 cm) were domesticated in aerated tap water for 24 h, fed once daily for seven consecutive days, and stopped being fed before the experiment.

### 3.6. Observation of Cellular Morphology

There were morphological changes of algae cells treated by bacillamide A, BDs, and the positive control (CuSO_4_). Scanning electron microscopy (SEM) and an optical microscope were used to detect the morphological changes of treated algae cells, after treatment of bacillamide A, BDs, and the positive control (CuSO_4_) at 10 mg/L for 4 h. Cell samples were centrifuged at 12,000 rpm for 10 min, collected, fixed, gradient-dehydrated, and freeze-dried after gold coating following the reported procedure [26]. The SEM analyses were performed by scanning electron microscopy (SEM, JSM-7900F, Hitachi High-Tech Corporation, Tokyo, Japan).

### 3.7. Determination of the Indicators of the Antioxidant System and the Photosynthetic System

*P. minimum*, *S. costatum*, and *A. pacificum* cells were cultured in F/2 medium. All flasks were cultivated under 12 h of light: 12 h of dark and 70 μmol m^−2^ s^−1^ at 20 ± 1 °C. Algae solution (20 mL) was exposed to **6j**, bacillamide A, CuSO_4_, and DMSO at concentrations of 10 mg/L, followed by centrifuge at 4000 r/min for 10 min. Finally, the algal cells were collected and diluted in 6 mL of phosphate buffer (pH = 7.8). A small amount of quartz sand was added into the algal cells under ice bath. The obtained homogenate was next poured into a centrifuge tube and centrifuged at 5000 r/min for 25 min at 4 °C. The obtained supernatant was placed in the refrigerator at 4 °C for the next step experiment.

Fv/Fm values were determined by using AquaPen handheld algae fluorescence meter (Nanjing Mingao Instrument Co., Ltd., Nanjing, China). The algae cells were darkened for 30 min and Fv/Fm values were measured.

The reactive oxygen species (ROS) assay kit was purchased from SolarBio Co., Ltd., Beijing, China. The 2′,7′-dichlorodihydrofluorescein diacetate (DCFH-DA) could be oxidized by reactive oxygen or nitrogen species (ROS/RNS) to generate high fluorescent 2′,7′-dichlorofluoroscein (DCF). DCF could be detected by Cyto-FLEX flow cytometry. The ROS values were measured using a fluorescent probe assay in algae treatment with or without **6j**, bacillamide A, CuSO_4_, and DMSO.

The malondialdehyde (MDA) assay kit was purchased from Lvyuandade Biochemical Co, Ltd., Beijing, China. The MDA parameter was monitored according to the procedure provided by the manufacturer. A total of 1 mL of algal extract was mixed with thiobarbituric acid solution (2 mL, 0.6%). The solution was sealed and kept in a boiling water bath for 15 min, followed by being cooled and centrifuged at 4500 r/min for 10 min. The absorbance values of collected supernatant were measured and calculated by the Formula (3) at 600 nm, 532 nm, and 450 nm:

MDA content (nmol∙mgprot^−1^) = 6.45 × (A_532_ − A_600_) − 0.56 × A_450_ (3)

### 3.8. Data Analysis

All experiment results were performed in triplicates unless otherwise specified. Results have been expressed as mean ± standard deviation (SD). Statistical analyses were conducted with SPSS. The differences were considered significant at *p* < 0.05. Graphs were plotted using Origin 2022 and Graph Pad Prism.

## 4. Conclusions

In summary, inspired by the natural algicide bacillamide A, 17 thiazole-containing derivatives were designed and synthesized, and their efficacy against three HAB species (*P. minimum*, *S. costatum*, and *A. pacificum*) was evaluated. Compound **6j** displayed comparable or superior algicidal activity to Diuron and CuSO_4_. Preliminary evaluations into the algacidal mechanism of **6j** illustrated that it can reduce the Fv/Fm of PSII and increase ROS and MDA levels in algal cells, but its algicidal effect was weaker than that of CuSO_4_. Bacillamide A and **6j** inhibited algal growth through different mechanisms than CuSO_4_. Unfortunately, although bacillamide A was less toxic to zebrafish (low toxicity) than CuSO_4_ (moderate toxicity), the acute toxicity of **6j** was classified as moderate. These findings suggest that both bacillamide A and **6j** have the potential to serve as lead candidates for the development of highly effective, environmentally friendly algicidal agents for controlling HABs.

## Figures and Tables

**Table 1:** The EC_50_ values of selected BDs against *S. costatum*, *P. minimum*, and *A. pacificum*.

N°	Compound	EC_50_ (μg/mL)		
		*S. costatum*	*A* *. pacificum*	*P* *. minimum*
**6a**	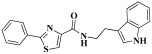	0.11 ± 0.02	3.32 ± 0.79	1.74 ± 0.40
**6c**	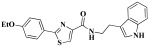	0.51 ± 0.04	2.43 ± 0.42	1.29 ± 0.08
**6j**	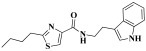	0.30 ± 0.02	0.47 ± 0.25	1.00 ± 0.11
**7b**	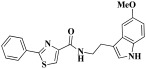	0.37 ± 0.07	0.72 ± 0.021	1.32 ± 0.38
**7c**	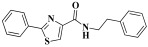	0.24 ± 0.058	0.53 ± 0.32	3.38 ± 0.84
**7d**	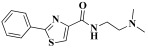	0.77 ± 0.21	2.02 ± 0.54	3.22 ± 0.39
**7e**	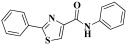	1.10 ± 0.31	0.69 ± 0.07	3.23 ± 0.39
**7g**	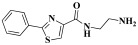	2.28 ± 0.37	3.42 ± 0.13	2.02 ± 0.27
bacillamide A	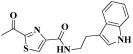	2.51 ± 0.09	2.17 ± 0.31	3.93 ± 0.28
Diuron	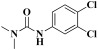	4.32 ± 0.14	5.69 ± 0.46	4.72 ± 0.41
CuSO_4_	CuSO_4_	3.12 ± 0.21	2.34 ± 0.06	3.61 ± 0.32

The full structure and the EC_50_ values of BDs are presented. Mean ± SD, n = 3.

## Data Availability

Data are contained within the article and Supplementary Materials.

## Supplementary Materials

Additional experimental procedures, characterization of BDs **6a**–**6k**, **7b**–**7f**, the ^1^H, and ^13^C NMR spectra of **6a**–**6k**, **7b**–**7g**, and bacillamide A in JEPG format (JEPG). The following supporting information can be downloaded at https://www.mdpi.com/article/10.3390/md22110494/s1, Table S1.
