# Supplementary material for: Design, Synthesis, and Evaluation of Novel Thiazole-Containing Algicides Inspired by Bacillamide A
Source: Mar Drugs. 2024 Nov 1;22(11):494. doi: 10.3390/md22110494 (PMC11595864; doi:10.3390/md22110494)

Design, Synthesis, and Evaluation of Novel Thiazole-containing Algicides Inspired by Bacillamide A

Xiaoxue Li[1&], Huili Li[2&], Lei Shi[1&], Zuguang Yin[1], Yuguo Du[3], Hongxia Zhang[2], Xin Wang[2], Xinxin Wang[2], Kexin Xu[2], Weili Wang[4], Ronglian Xing[2*], Yi Liu[1*],

[1] School of Chemistry and Chemical Engineering, Yantai University, Yantai 264005, China;

[2] School of Life Sciences, Yantai University, Yantai, Shandong Province, 264005, P.R.China;

[3] State Key Laboratory of Environmental Chemistry and Eco-toxicology, Research Center for Eco-Environmental Sciences, Chinese Academy of Sciences, Beijing 100085, China;

[4] School of Chemistry and Material Science, Ludong University, 186 Hongqi Middle Road, Yantai, 264025, China;

[*] Corresponding author.

[&] These authors contributed equally to this work.

Authors to whom correspondence should be addressed at xingronglian@163.com and liuyi@ytu.edu.cn

Contents

1. General experimental details S3

2. Characterization of BDs 6a-6k, 7b-7f...S3

3. Reference S11

4. Acute toxicity table S1. ....S12

5. Copies of NMR Spectra..**S13**

1. General Experimental Details

Unless other indicated, all reagents were purchased from commercial corporations. All reactions were performed in oven-dried glassware under standard conditions. Flash chromatography (FC) was performed using silica gel (200-300 meshes). High-resolution mass spectrometry data were acquired using a Q-TOF analyzer. Optical rotations were recorded on a Rudolph Polarimeter Autopol 111. Melting points were analyzed on a Melt-Temp II capillary melting point apparatus. ^{1}H NMR, ^{13}C NMR and HMBC were measured on 400 MHz/100 MHz or 500 MHz/125 MHz Bruker spectrometers or JEOL JNM-ECZ400S spectrometers (NMR in $CDCl_3$ with TMS as an internal standard). Chemical shifts (δ) are given in ppm relative to residual solvent (usually $CDCl_3$: δ 7.26 for ^{1}H NMR or 77.23 for proton decoupled ^{13}C NMR; DMSO-d_6; δ 2.50 for ^{1}H NMR or 39.53 for proton decoupled ^{13}C NMR; CD_3OD; δ 3.31 for ^{1}H NMR or 49.03 for proton decoupled ^{13}C NMR), and coupling constants (J) in Hz. Signals are reported as follows: s (singlet), d (doublet), t (triplet), q (quartet), dd (doublet of doublets), dt (doublet of triplets), ddt (doublet of doublets of triplets), dq (doublet of quartets), br s (broad singlet), m (multiplet). β-azido disulfide **2** and thiazole methyl ester **3a-3k** was prepared according to our previous report[1].

2. Characterization of BDs 6a-6k, 7b-7f

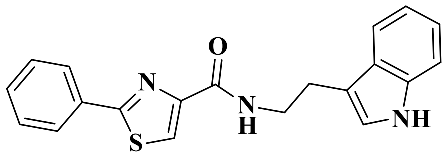

N-(2-(1H-indol-3-yl)ethyl)-2-phenylthiazole-4-carboxamide (6a) Following the synthetic route A gave **6a** (eluent: PET : EA = 2:1, Rf = 0.50) as white solid (67% for three steps); m.p.: 182-183 °C; ^{1}H NMR (400 MHz, $CDCl_3$) δ: 8.24 (s, 1H), 8.09 (s, 1H), 7.89-7.85 (m, 2H), 7.70 (d, J = 8.0 Hz, 1H), 7.65-7.61 (m, 1H), 7.44-7.60 (m, 3H), 7.40 (d, J = 8.0 Hz, 1H), 7.24 (t, J = 7.6 Hz, 1H), 7.14 (t, J = 7.6 Hz, 1H), 7.11 (s, 1H), 3.83 (dt, J = 6.4 Hz, J = 13.2 Hz, 2H), 3.14 (t, J = 6.4 Hz, 2H); ^{13}C NMR (100 MHz, $CDCl_3$) δ:168.2, 161.3, 151.1, 136.6, 133.0, 130.8, 129.2, 127.6, 126.8,

122.9, 122.3, 119.7, 119.0, 113.3, 111.4, 40.1, 25.7; ESI-HRMS calcd for $C_{20}H_{17}N_3NaOS$ ([M + Na]$^+$) 370.0990, found 370.0994.

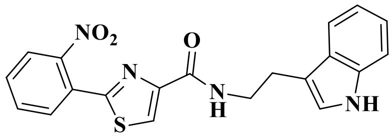

N-(2-(1H-indol-3-yl)ethyl)-2-(2-nitrophenyl)thiazole-4-carboxamide (6b)

Following the synthetic route A gave **6b** (eluent: PET : EA = 2:1, Rf = 0.50) as white solid (60% for three steps); m.p.: 122-123 °C; ^{1}H NMR (400 MHz, $CDCl_3$): δ 8.28 (s, 1H), 8.22 (s, 1H), 7.85 (d, J = 7.6 Hz, 1H), 7.86-7.60 (m, 4H), 7.40 (d, J = 8.0 Hz, 1H), 7.34 (s, 1H), 7.20 (t, J = 7.6 Hz, 1H), 7.18-7.10 (m, 2H), 3.73 (dt, J = 6.4 Hz, J = 12.4 Hz, 2H), 3.07 (t, J = 6.4 Hz, 2H); ^{13}C NMR (100 MHz, $CDCl_3$) δ 162.1, 160.6, 151.6, 148.7, 136.7, 132.6, 131.6, 131.1, 127.3, 126.8, 124.9, 124.8, 123.0, 122.2, 119.5, 118.9, 112.7, 111.5, 39.4, 25.5. ESI-HRMS calcd for $C_{20}H_{16}N_4NaO_3S$ ([M + Na]$^+$) 415.0835, found 415.0826;

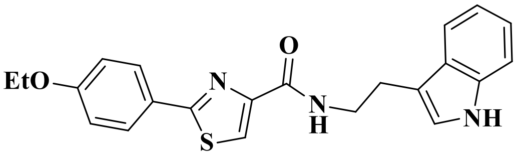

N-(2-(1H-indol-3-yl)ethyl)-2-(4-ethoxyphenyl)thiazole-4-carboxamide (6c)

Following the synthetic route A gave **6c** (eluent: PET : EA = 2:1, Rf = 0.40) as white solid (63% for three steps); m.p.: 184-185 °C; ^{1}H NMR (400 MHz, $CDCl_3$) δ: 8.41 (s, 1H), 8.01 (s, 1H), 7.78 (d, J = 8.0 Hz, 2H), 7.75-7.60 (m, 2H), 7.40 (d, J = 8.0 Hz, 1H), 7.23 (t, J = 7.5 Hz, 1H), 7.14 (t, J = 7.5 Hz, 1H), 7.09 (s, 1H), 6.93 (d, J = 8.0 Hz, 2H), 4.08 (q, J = 7.8 Hz, 2H), 3.82 (q, J = 6.8 Hz, 2H), 3.13 (t, J = 6.8 Hz, 2H), 1.45 (t, J = 6.8 Hz, 3H); ^{13}C NMR (100 MHz, $CDCl_3$) δ: 167.6, 160.9, 160.5, 150.2, 136.0, 127.7, 127.0, 125.2, 121.8, 121.6, 121.4, 119.0, 118.4, 114.4, 112.6, 110.9, 63.3, 39.6, 25.0, 14.3. ESI-HRMS calcd for $C_{22}H_{21}N_3NaO_2S$ ([M + Na]$^+$) 414.1252, found 414.1259.

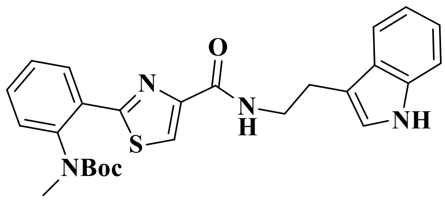

tert-butyl(2-(4-((2-(1H-indol-3-yl)ethyl)carbamoyl)thiazol-2-yl)phenyl)carbamate (6d) Following the synthetic route A gave **6d** (eluent: PET : EA = 2:1, Rf = 0.55) as white solid (57% for three steps); white powder; ^{1}H NMR (400 MHz, $CDCl_3$) δ: 8.41 (s, 1H), 8.17 (s, 1H), 7.97 (t, J = 8.4 Hz, 2H), 7.74 (d, J = 8.4 Hz, 1H), 7.48 (t, J = 7.2 Hz, 1H), 7.45-7.38 (m , 2H), 7.30-7.25(m, 1H)，7.23 (t, J = 7.2 Hz, 1H), 7.15 (t, J = 7.2 Hz, 2H), 3.87 (q, J = 7.2 Hz, 2H), 3.20-3.14 (m, 5H), 1.19 (s, 9H); ^{13}C NMR (100 MHz, $CDCl_3$) δ 164.1, 161.2, 155.4, 150.5, 141.2, 136.3, 131.0, 129.9, 129.1, 129.0, 127.7, 127.5, 123.4, 122.1, 122.0, 119.4, 118.9, 113.2, 111.1, 80.3, 39.9, 36.7, 28.0, 25.5. $C_{26}H_{29}N_4O_3S$ ([M + H]$^+$) 477.1960, found 477.1965.

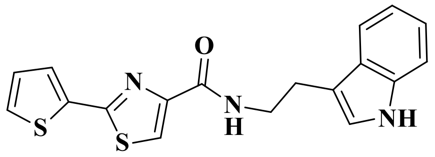

N-(2-(1H-indol-3-yl)ethyl)-2-(thiophen-2-yl)thiazole-4-carboxamide (6e) Following the synthetic route A gave **6e** (eluent: PET : EA = 2:1, Rf = 0.50) as white solid (62% for three steps); data are consistent with a previously characterized compound[2, 3].m.p. : 189-190 °C; ^{1}H NMR (400 MHz, $CDCl_3$) δ: 8.22 (br s, 1H), 8.01 (s, 1H), 7.69 (d, J = 7.6 Hz, 1H), 7.56 (s, 1H), 7.50 (d, J = 1.6 Hz, 1H), 7.43-7.38 (m, 2H), 7.22 (t, J = 7.6 Hz, 1H), 7.16-7.08 (m, 3H), 3.79 (dt, J = 6.0 Hz, J = 13.2 Hz, 2H), 3.18 (t, J = 7.2 Hz, 2H). ^{13}C NMR (100 MHz, $CDCl_3$) δ 161.7, 161.0, 150.6, 136.6, 136.5, 128.6, 128.2, 127.6, 127.5, 122.3, 122.3, 119.7, 119.0, 113.2, 111.4, 39.9, 25.7. $C_{18}H_{15}N_3NaO_2S$ ([M + Na]$^+$) 376.0549, found 376.0543.

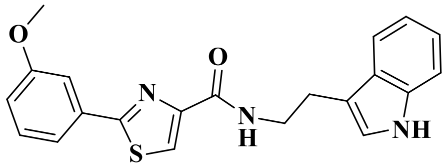

N-(2-(1H-indol-3-yl)ethyl)-2-(3-methoxyphenyl)thiazole-4-carboxamide (6f) Following the synthetic route A gave **6f** (eluent: PET : EA = 2:1, Rf = 0.45) as white solid (62% for three steps); colorless oil; ^{1}H NMR (400 MHz, $CDCl_3$) δ: 8.22 (br s, 1H), 8.09 (s, 1H), 7.69 (d, J = 7.6 Hz, 1H), 7.65-7.60 (m, 1H), 7.43 (s, 1H), 7.38 (t, J = 7.6 Hz, 2H), 7.35 (t, J = 7.6 Hz, 2H), 7.22 (t, J = 7.6 Hz, 1H), 7.14 (t, J = 7.6 Hz, 1H), 7.11 (s, 1H), 7.00 (dd, J = 1.2 Hz, J = 8.0 Hz, 1H), 3.87 (s, 3H), 3.82 (dt, J = 6.8 Hz, J =13.2 Hz, 2H), 3.13 (t, J = 6.8 Hz, 2H). ^{13}C NMR (100 MHz, $CDCl_3$) δ 168.1, 161.4, 160.3, 151.1, 136.6, 134.3, 130.4, 127.6, 123.1, 122.4, 119.7, 119.5, 119.0, 116.5, 113.3, 112.1, 111.4, 55.7, 40.0, 25.8. $C_{21}H_{19}N_3NaO_2S$ ([M + Na]$^+$) 400.1090, found 400.1083.

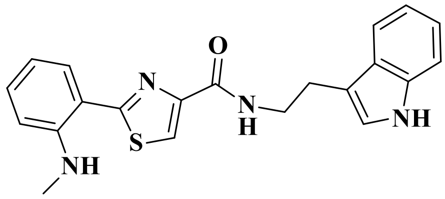

N-(2-(1H-indol-3-yl)ethyl)-2-(2-(methylamino)phenyl)thiazole-4-carboxamide (6g) Following the synthetic route A gave **6g** (eluent: PET : EA = 2:1, Rf = 0.40) as colorless oil (61% for three steps); ^{1}H NMR (400 MHz, $CDCl_3$) δ: 8.45 (br s, 1H), 8.00 (s, 1H), 7.66 (d, J = 8.0 Hz, 1H), 7.60 (dd, J = 1.2 Hz, J = 8.0 Hz, 1H), 7.38 (dd, J = 1.2 Hz, J = 8.0 Hz, 1H), 7.31 (dt, J = 1.2 Hz, J = 6.8 Hz, 1H), 7.23-7.19 (m, 2H), 7.12 (dt, J = 1.2 Hz, J = 6.8 Hz, 1H), 7.06 (d, J = 3.6 Hz, 1H), 6.71-6.66 (m, 2H), 3.85 (dd, J = 6.4 Hz, J = 12.6 Hz, 2H), 3.11 (d, J = 6.4 Hz, 2H), 2.72 (s, 3H); ^{13}C NMR (100 MHz, $CDCl_3$) δ 170.1, 161.2, 149.9, 147.1, 136.7, 132.1, 129.9, 127.4, 122.4, 122.4，121.6, 119.7, 118.9, 115.9, 114.9, 112.9, 111.5, 111.3, 39.6, 30.0, 25.7. $C_{21}H_{20}N_4NaOS$ ([M + Na]$^+$) 399.1250, found 399.1245.

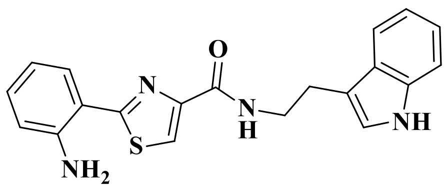

N-(2-(1H-indol-3-yl)ethyl)-2-(2-aminophenyl)thiazole-4-carboxamide (6h)

Following the synthetic route A gave **6h** (eluent: PET : EA = 2:1, Rf = 0.45) as powder (47% for three steps) as colorless oil; ^{1}H NMR (400 MHz, $CDCl_3$) δ: 8.83 (t, J = 6.0 Hz, 1H), 8.18 (s, 1H), 7.63 (d, J = 8.0 Hz, 1H), 7.58 (d, J = 8.0 Hz, 1H), 7.34 (d, J = 8.0 Hz, 1H), 7.19 (s, 1H), 7.17 (t, J = 8.0 Hz, 1H), 7.07 (t, J = 8.0 Hz, 1H), 6.99 (t, J = 7.2 Hz, 1H), 6.91-6.75 (m, 3H), 6.60 (t, J = 7.2 Hz, 1H), 3.60-3.55 (m, 2H), 2.98 (t, J = 7.6 Hz, 2H). ^{13}C NMR (100 MHz, $CDCl_3$) δ 169.0, 160.4, 150.4, 146.4, 136.3, 131.1, 129.2, 127.2, 122.5, 121.6, 121.0, 118.4, 118.3, 116.7, 115.5, 113.4, 111.8, 111.4, 25.4. $C_{20}H_{18}N_4NaOS$ ([M + Na]$^+$) 385.1094, found 385.1102.

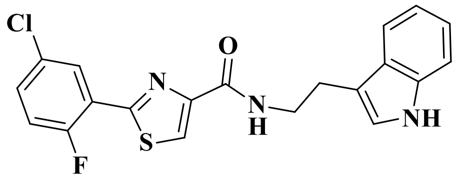

N-(2-(1H-indol-3-yl)ethyl)-2-(5-chloro-2-fluorophenyl)thiazole-4-carboxamide (6i) Following the synthetic route A gave **6i** (eluent: PET : EA = 2:1, Rf = 0.50) as white solid (61% for three steps); m.p.: 213-215 °C; ^{1}H NMR (500 MHz, DMSO-d_6) δ: 8.90 (t, J = 6.0 Hz, 1H), 8.53-8.51 (m, 1H), 8.45 (s, 1H), 7.65-7.61 (m, 2H), 7.54-7.50 (m, 1H), 7.34 (d, J = 8.0 Hz, 1H), 7.22 (s, 1H), 7.07 (t, J = 7.5 Hz, 1H), 3.62 (dd, J = 6.0 Hz, J = 15.0 Hz, 2H), 3.01 (t, J = 6.0 Hz, 2H). ^{13}C NMR (125 MHz, DMSO-d_6) δ: 162.7, 161.2 (d, J = 164.5 Hz), 160.0 (d, J = 79.5 Hz), 152.5, 134.2 (d, J = 9.0 Hz), 131.9, 130.3, 129.8, 128.3 (d, J = 9.0 Hz), 125.1, 124.2 (d, J = 14.0 Hz), 123.5, 120.9 (d, J = 7.5 Hz), 120.8, 114.3, 113.9, 42.3, 27.9. $C_{20}H_{15}ClFN_3NaOS^+$ ([M + Na]$^+$) 422.0501, found 422.0516.

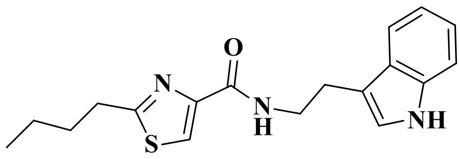

N-(2-(1H-indol-3-yl)ethyl)-2-butylthiazole-4-carboxamide (6j) Following the synthetic route A gave **6j** (eluent: PET : EA = 2:1, Rf = 0.45) as white solid (51% for three steps); m.p.: 174-177 °C; ^{1}H NMR (500 MHz, $CDCl_3$) δ: 8.12 (s, 1H), 7.96 (s, 1H), 7.66 (dd, J = 1.0 Hz, J = 8.0 Hz, 1H), 7.51-7.46 (m, 1H), 7.38 (dd, J = 1.0 Hz, J = 8.0 Hz, 1H), 7.21 (dt, J = 1.0 Hz, J = 8.0 Hz, 1H), 7.13 (dt, J = 1.0 Hz, J = 6.0 Hz, 1H), 7.08 (s, J = 2.5 Hz, 1H), 3.78 (q, J = 7.0 Hz, 2H), 3.10 (t, J = 7.0 Hz, 2H), 2.94 (t, J = 8.0 Hz, 2H), 1.74 (ddt, J = 2.0 Hz, J = 8.0 Hz, J = 15.0 Hz, 2H), 1.42 (ddt, J = 2.0 Hz, J = 8.0 Hz, J = 15.0 Hz, 2H), 0.96 (t, J = 8.0 Hz, 3H). ^{13}C NMR (125 MHz, $CDCl_3$) δ 171.7, 161.6, 149.9, 136.6, 127.6, 122.4, 122.4, 122.2, 119.7, 119.1, 113.4, 111.4, 39.9, 33.2, 32.1, 25.8, 22.4, 14.0. $C_{18}H_{22}N_3OS$ ([M + H]$^+$) 328.1478, found 328.1487.

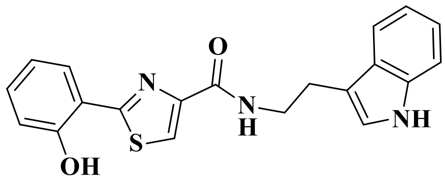

N-(2-(1H-indol-3-yl)ethyl)-2-(2-hydroxyphenyl)thiazole-4-carboxamide (6k) Following the synthetic route A gave **6k** (eluent: PET : EA = 1:1, Rf = 0.55) as colorless oil (45% for three steps); ^{1}H NMR (400 MHz, CD_3OD) δ: 8.15 (s, 1H), 8.03 (dd, J = 1.2 Hz, J = 8.0 Hz, 1H), 7.65 (d, J = 8.0 Hz, 1H), 7.35 (d, J = 8.0 Hz, 2H), 7.33 (t, J = 10.0 Hz, 1H), 7.15 (s, 1H), 7.09 (t, J = 8.0 Hz, 2H), 7.02-6.94 (m, 1H), 3.73 (t, J = 7.2 Hz, 2H), 3.11 (t, J = 7.2 Hz, 2H). ^{13}C NMR (100 MHz, DMSO-d_6) δ 162.7, 160.6, 155.0, 148.9, 136.3, 131.3, 128.0, 127.3, 123.6, 122.6, 121.0, 119.4, 119.1, 118.4, 118.3, 116.4, 111.8, 111.4, 25.3. $C_{20}H_{18}N_3O_2S$ ([M + H]$^+$) 364.1114, found 364.1111.

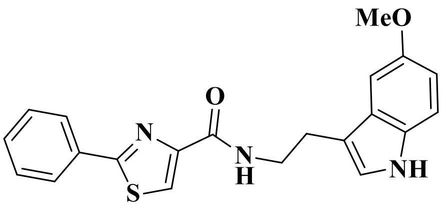

N-(2-(5-methoxy-1H-indol-3-yl)ethyl)-2-phenylthiazole-4-carboxamide (7b) Following the synthetic route B gave **7b** (eluent: PET : EA = 1:1, Rf = 0.45) as white solid (45% from carboxyl acid); m.p.: 164-165 °C;_^{1}H NMR (400 MHz, CD_3OD) δ: 8.15 (s, 1H), 8.03 (dd, J = 1.2 Hz, J = 8.0 Hz, 1H), 7.65 (d, J = 8.0 Hz, 1H), 7.35 (d, J = 8.0 Hz, 1H), 7.33 (t, J = 10.0 Hz, 1H), 7.15 (s, 1H), 7.09 (t, J = 8.0 Hz, 1H), 7.02-6.94 (m, 2H), 3.73 (t, J = 7.2 Hz, 2H), 3.11 (t, J = 7.2 Hz, 2H). ^{13}C NMR (100 MHz, DMSO-d_6) δ 162.7, 160.6, 155.0, 148.9, 136.3, 131.3, 128.0, 127.3, 123.6, 122.6, 121.0, 119.4, 119.1, 118.4, 118.3, 116.4, 111.8, 111.4, 25.3. $C_{20}H_{30}N_3O_2S$ ([M + H]$^+$) 378.1271, found 378.1271.

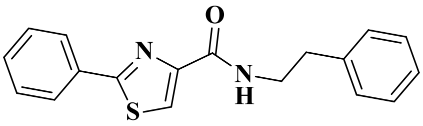

N-phenethyl-2-phenylthiazole-4-carboxamide (7c) Following the synthetic route B gave **7c** (eluent: PET : EA = 1:1, Rf = 0.45) as colorless oil (45% from carboxyl acid); ^{1}H NMR (500 MHz, $CDCl_3$) δ: 8.02 (s, 1H), 7.81-7.77 (m, 2H), 7.50 (t, J = 6.0 Hz, 1H), 7.35-7.30 (m, 3H), 7.24-7.21 (m, 2H), 7.17-7.13 (m, 3H), 3.62 (dd, J = 8.0 Hz, J = 15.0 Hz, 2H), 2.84 (t, J = 8.0 Hz, 2H); ^{13}C NMR (100 MHz, $CDCl_3$) δ: 168.1, 161.2, 150.9, 139.0, 132.9, 130.7, 129.2, 129.0, 128.8, 126.7, 126.7, 122.9, 40.8, 36.1. $C_{18}H_{16}N_2NaO_3S^+$ ([M + Na]$^+$) 309.1056, found 309.1053.

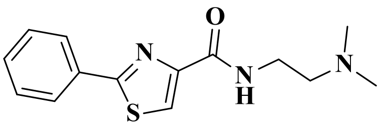

N-(2-(dimethylamino)ethyl)-2-phenylthiazole-4-carboxamide (7d) Following the synthetic route B gave **7d** (eluent: PET : EA = 1:1, Rf = 0.30) as white solid (45%

from carboxyl acid); m.p.: 133-134 °C; ^{1}H NMR (500 MHz, $CDCl_3$) δ: 8.08 (s, 1H), 7.97-7.94 (m, 2H), 7.96 (br s, 1H), 7.48-7.45 (m, 3H), 3.57 (dt, J = 6.0 Hz, J = 12.0 Hz, 2H), 2.55 (t, J = 6.0 Hz, 2H), 2.30 (s, 6H); ^{13}C NMR (100 MHz, $CDCl_3$) δ: 168.3, 161.5, 151.2, 133.2, 130.8, 129.3, 126.9, 123.0, 58.5, 45.7, 37.3. $C_{14}H_{17}N_3NaOS^+$ ([M + Na]$^+$) 298.0985, found 298.0977.

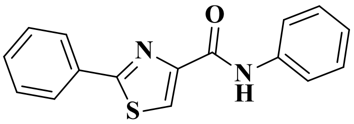

N,2-diphenylthiazole-4-carboxamide (7e) Following the synthetic route B gave **7e** (eluent: PET : EA = 2:1, Rf = 0.60) as colorless oil (45% from carboxyl acid); ^{1}H NMR (500 MHz, $CDCl_3$) δ: 9.31 (s, 1H), 8.20 (s, 1H), 8.01-7.98 (m, 2H), 7.77-7.50 (m, 2H), 7.51-7.48 (m, 3H), 7.40 (t, J = 8.5 Hz, 2H), 7.18-7.15 (m, 1H); ^{13}C NMR (100 MHz, $CDCl_3$) δ: 168.5, 159.1, 151.1, 137.9, 132.9, 131.0, 129.4, 129.3, 126.9, 124.7, 123.9, 120.1. $C_{16}H_{12}N_2NaOS^+$ ([M + Na]$^+$) 303.0563, found 303.0547.

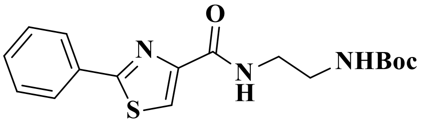

tert-butyl (2-(2-phenylthiazole-4-carboxamido)ethyl)carbamate (7f) Following the synthetic route B gave **7f** (eluent: PET : EA = 2:1, Rf = 0.60) as white solid (45% from carboxyl acid); m.p. : 162-163 °C; ^{1}H NMR (500 MHz, $CDCl_3$) δ: 8.07 (s, 1H), 7.96-7.92 (m, 3H), 7.45-7.42 (m, 3H), 5.14 (s, 1H), 3.58 (dt, J = 6.0 Hz, J = 12.0 Hz, 2H), 3.40 (dt, J = 6.0 Hz, J = 12.0 Hz, 2H), 1.42 (s, 9H); ^{13}C NMR (100 MHz, $CDCl_3$) δ: 168.3, 162.1, 156.8, 150.7, 133.0, 130.8, 129.2, 126.9, 123.1, 79.8, 40.7, 40.4, 28.6. $C_{17}H_{21}N_3NaO_3S^+$ ([M + Na]$^+$) 37.1196, found 370.1196.

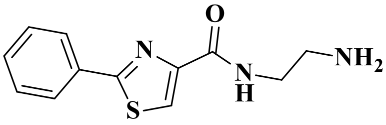

N-(2-aminoethyl)-2-phenylthiazole-4-carboxamide (7g) Following the synthetic route B gave **7g** (eluent: PET : EA = 2:1, Rf = 0.60) as white solid (45% from carboxyl acid); m.p. : 173-174 °C; ^{1}H NMR (500 MHz, DMSO-d_6) δ: 8.81 (t, J = 6.0 Hz, 1H), 8.34 (s, 1H), 8.10 (br s, 2H), 8.07-8.04 (m, 2H), 7.54-7.52 (m, 3H), 3.58 (dt, J = 6.0 Hz, J = 12.0 Hz, 2H), 3.04 (t, J = 6.0 Hz,2H); ^{13}C NMR (125 MHz, DMSO-d_6) δ: 167.2, 161.2, 150.4, 132.5, 130.8, 129.3, 126.5, 124.4, 38.7, 36.8. $C_{12}H_{13}N_3NaOS^+$ ([M + Na]$^+$) 270.0672, found 270.0667.

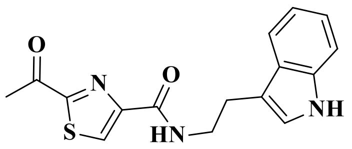

N-(2-(1H-indol-3-yl)ethyl)-2-acetylthiazole-4-carboxamide Following the reported procedure gave Bacillamide A (eluent: PET : EA = 1:1, Rf = 0.60) as colorless oil; ^{1}H NMR (500 MHz, $CDCl_3$) δ: 8.40 (s, 1H), 8.20 (br s, 1H), 7.67 (d, J = 8.0 Hz, 1H), 7.44-7.42 (m, 1H), 7.39 (d, J = 8.0 Hz, 1H), 7.21 (dt, J = 1.5 Hz, J = 8.0 Hz, 1H), 7.13 (dt, J = 1.0 Hz, J = 8.0 Hz, 1H), 7.10 (d, J = 2.5 Hz, 1H), 3.82 (q, J = 7.0 Hz, 2H), 3.13 (dt, J = 1.0 Hz, J = 7.0 Hz, 2H), 2.61 (s, 3H); ^{13}C NMR (125 MHz, $CDCl_3$) δ: 191.4, 166.6, 160.6, 151.9, 136.6, 129.8, 127.6, 122.5, 122.3, 119.8, 119.0, 113.2, 111.5, 40.2. 26.1, 25.6; $C_{16}H_{15}N_3NaO_2S^+$ ([M + Na]$^+$) 336.0777, found 336.0775.

3. Reference

1. Liu, Y.; Li, Z.; Xie, Y.; He, P.; Qiao, J.; Fan, X.; Du, Y., Efficient one-pot synthesis of 2, 4-disubstituted thiazoles and dimeric thiazoles directly from acyl chlorides and β-azido disulfides. *Synthesis* 2017, 49, (21), 4876-4886
2. Kumara, S.; Aggarwal, R., A concise and efficient route to the total synthesis of bacillamide A and its analogues. *Arkivoc.* **2018**, (part iii), 354-361.
3. Kumar, S.; Aggarwal, R.; Kumar, V.; Sadana, R.; Patel, B.; Kaushik, P.; Kaushik, D., Solvent-free synthesis of bacillamide analogues as novel cytotoxic and anti-inflammatory agents. *Eur. J. of Med. Chem.* **2016,** 123, 718-726.

4. Acute toxicity table S1.

Table S1. Acute toxicity of BDs (**6a** and **6j**), DMSO, $CuSO_4$, and bacillamide A in zebrafish.

Compound	Concentration (mg/L)	Survival percent					Acute toxicity
		0h	24h	48h	72h	96h	
	0.1	100	100	100	100	100	
DMSO	1	100	100	100	100	100	low
	10	100	90	90	80	70	
	0.1	100	100	100	100	100	
bacillamide A	1	100	100	87	83	83	low
	10	100	100	100	100	100	
	0.1	100	100	100	100	100	
**6a**	1	100	83	80	80	73	moderate
	10	100	0	0	0	0	
	0.1	100	100	100	100	100	
**6j**	1	100	100	100	80	80	moderate
	10	100	80	0	0	0	
	0.1	100	100	80	73	73	
$CuSO_4$	1	100	50	50	43	43	moderate
	10	100	0	0	0	0	

[a]According to the Guide for Environmental Safety Assessment of Chemical Pesticides (GB/T31270.12) ($LC_{50\ (96\ h)} \leq 0.1$ mg/L, high toxicity; $1.0 < LC_{50\ (96\ h)} \leq 10$ mg/L, moderate toxicity; $LC_{50\ (96\ h)} > 10$ mg/L, low toxicity), the zebrafish were devided into 4 groups and treated with different compounds for 96 h.

5.Copies of NMR Spectra

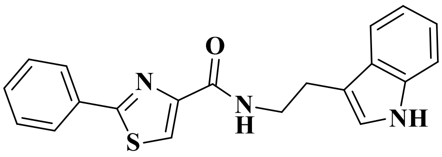

(6a)

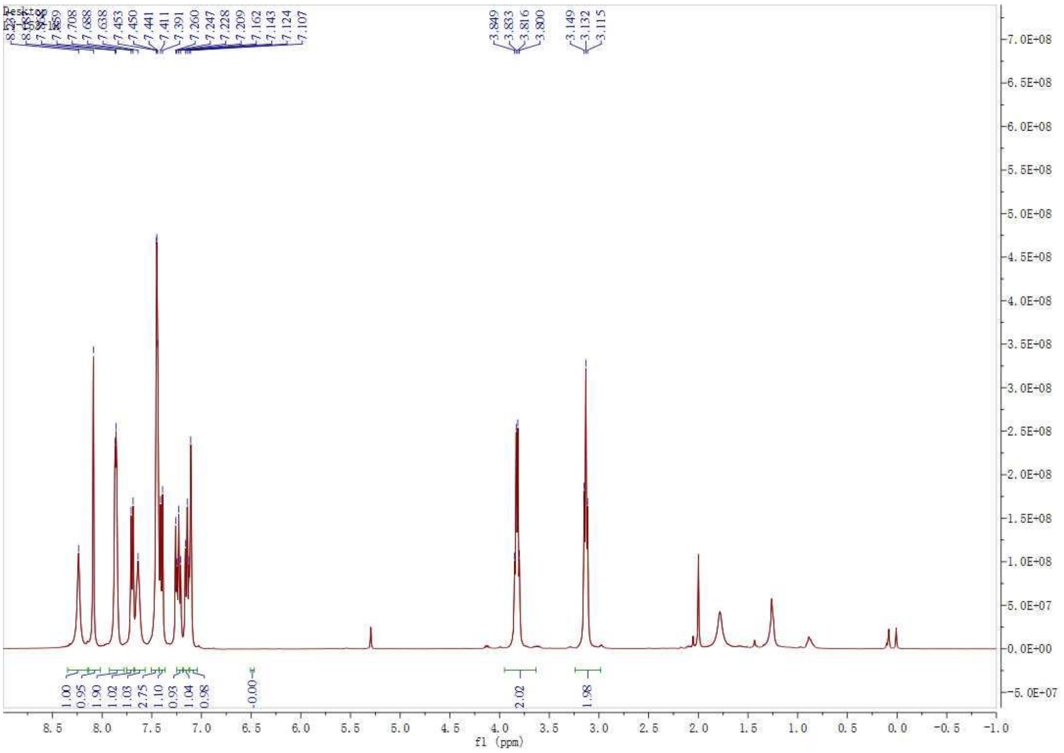

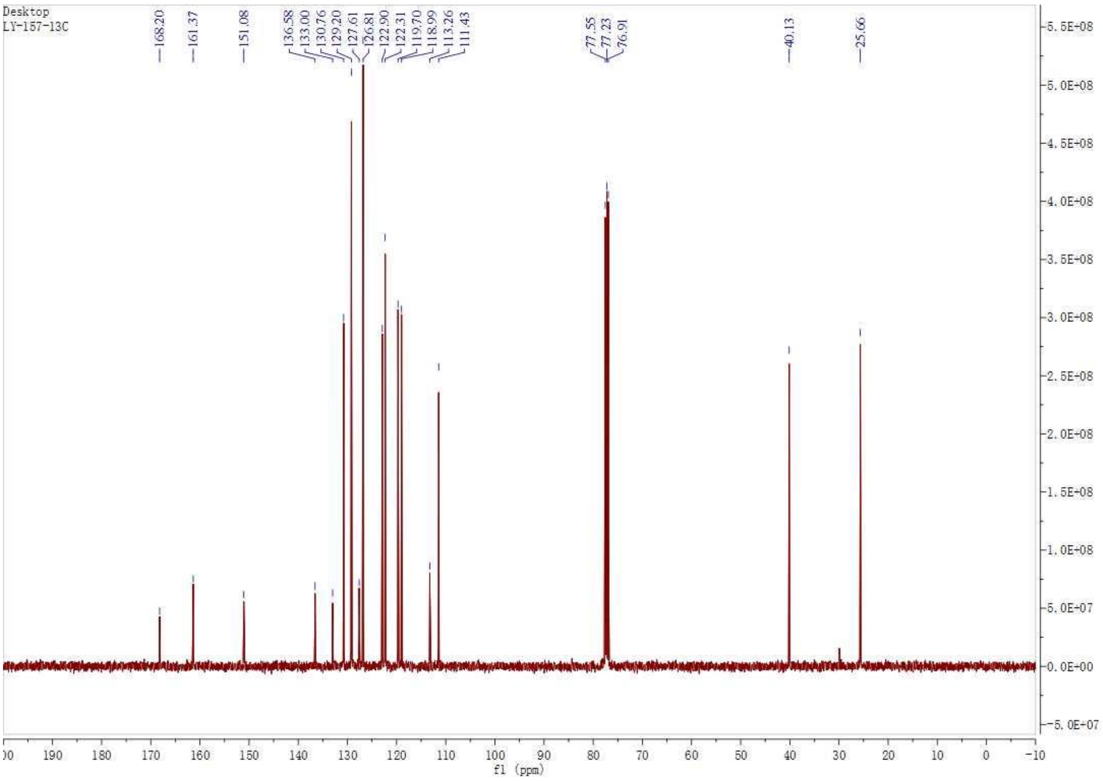

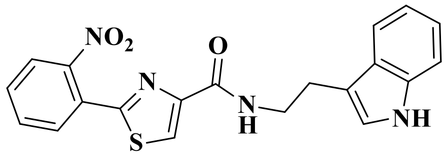
NO2
O
N
N
H
NH
S
(6b)

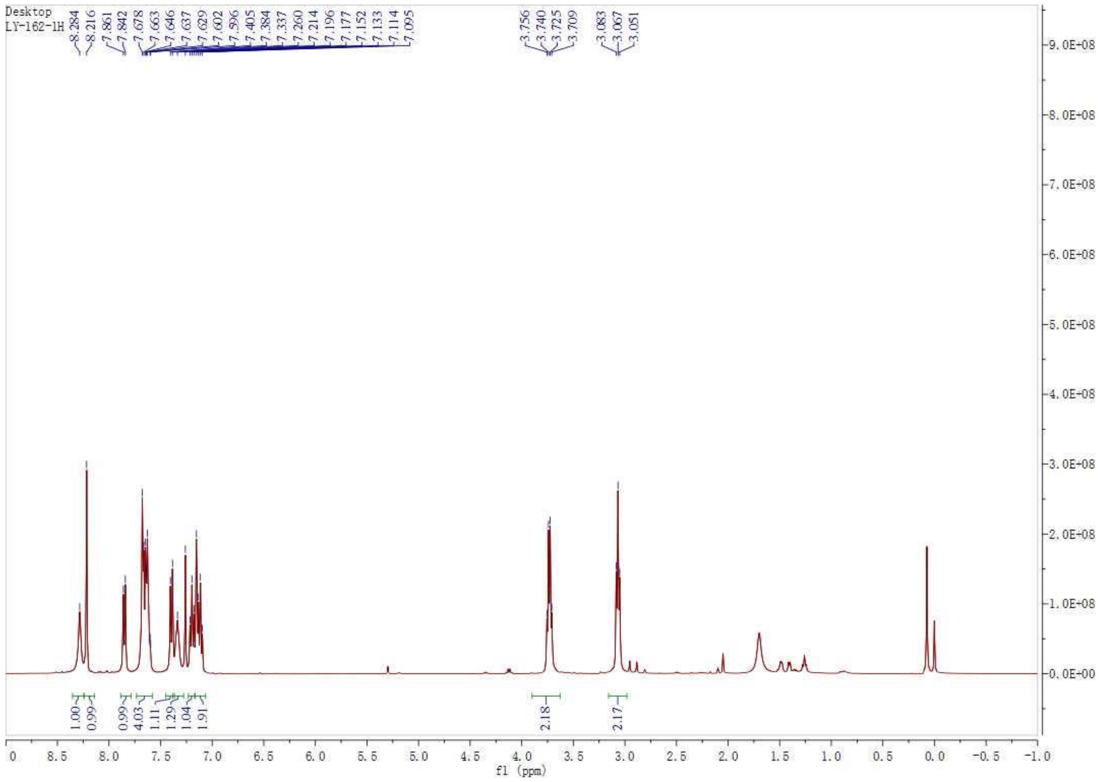
Desktop
LY-162-1H
8.284
8.216
7.861
7.842
7.678
7.663
7.646
7.637
7.629
7.602
7.596
7.405
7.384
7.337
7.260
7.214
7.196
7.177
7.152
7.133
7.114
7.095
3.756
3.740
3.725
3.709
3.083
3.067
3.051
1.00
0.99
0.99
4.03
1.11
1.29
1.04
1.91
2.18
2.17
f1 (ppm)

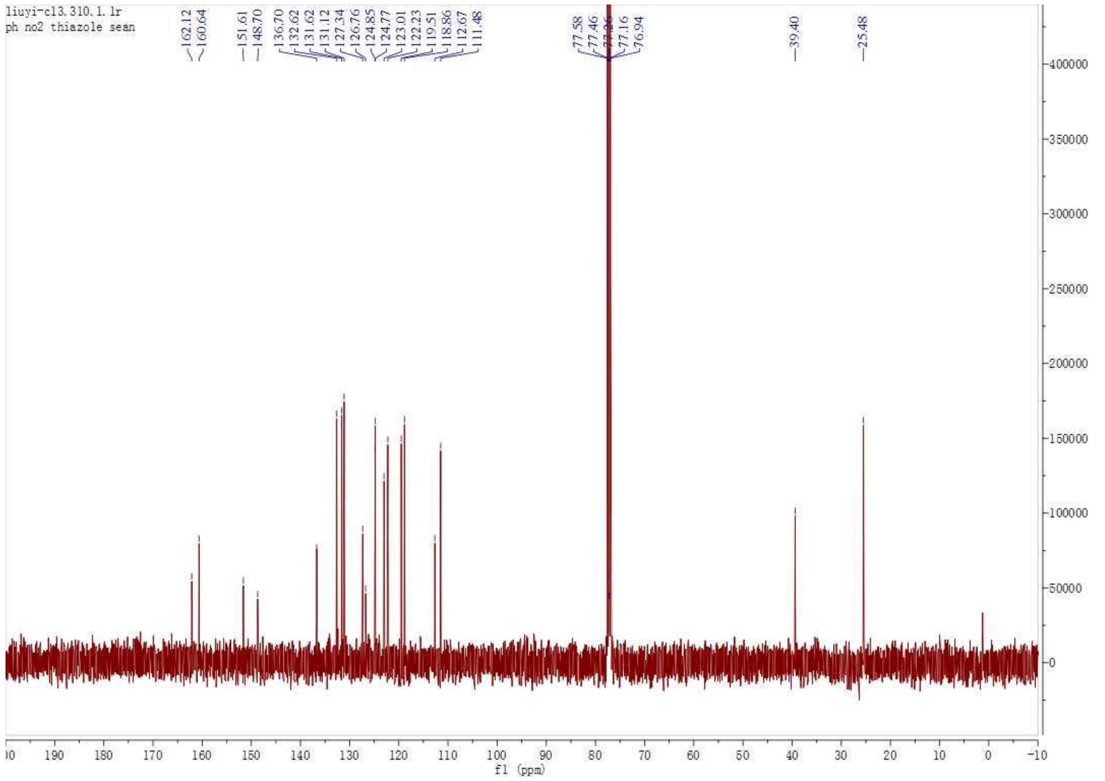
liuyi-c13.310.1.1r
ph no2 thiazole sean
162.12
160.64
151.61
148.70
136.70
132.62
131.62
131.12
127.34
126.76
124.85
124.77
123.01
122.23
119.51
118.86
112.67
111.48
77.58
77.46
77.16
76.94
39.40
25.48
f1 (ppm)

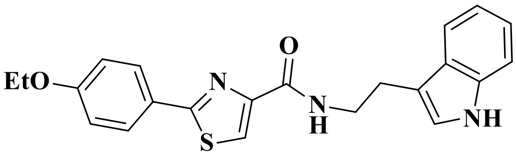
EtO
N
S
O
N
H
NH

(6c)

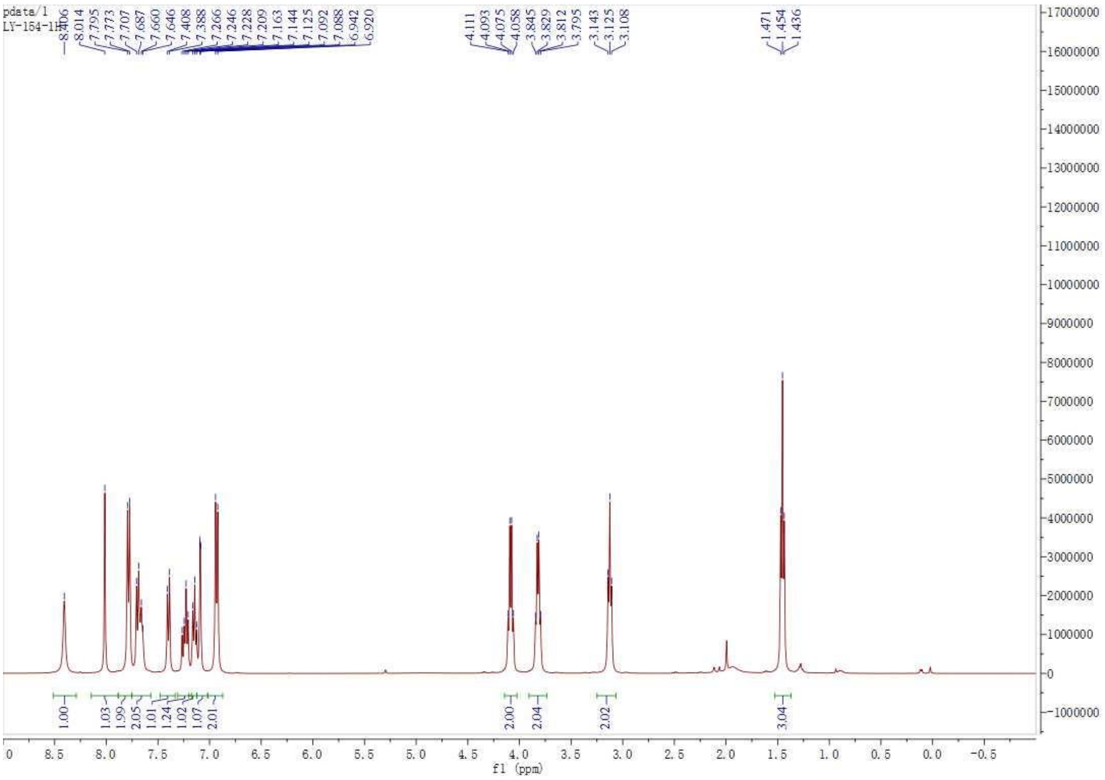
pdata/1
LY-154-1H
8.406
8.014
7.795
7.773
7.707
7.687
7.660
7.646
7.408
7.388
7.266
7.246
7.228
7.209
7.163
7.144
7.125
7.092
7.088
6.942
6.920
4.111
4.093
4.075
4.058
3.845
3.829
3.812
3.795
3.143
3.125
3.108
1.471
1.454
1.436
1.00
1.03
1.99
2.05
1.01
1.24
1.02
1.07
2.01
2.00
2.04
2.02
3.04
f1 (ppm)

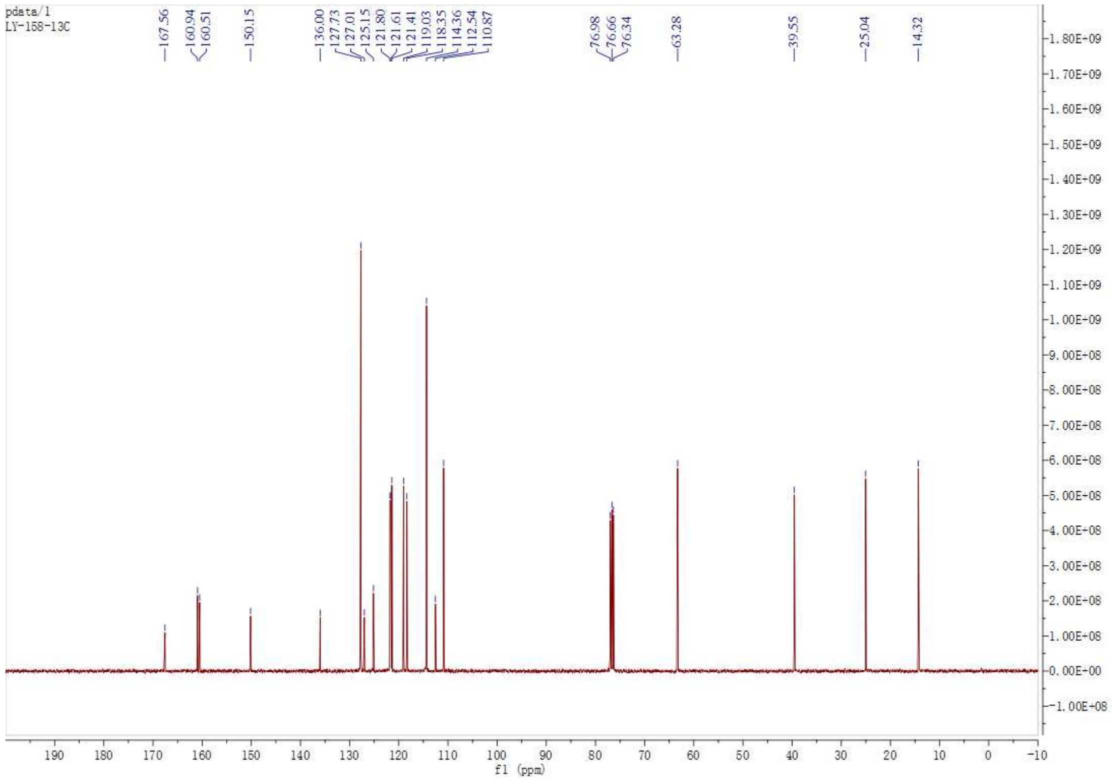
pdata/1
LY-158-13C
167.56
160.94
160.51
150.15
136.00
127.73
127.01
125.15
121.80
121.61
121.41
119.03
118.35
114.36
112.54
110.87
76.98
76.66
76.34
63.28
39.55
25.04
14.32
f1 (ppm)

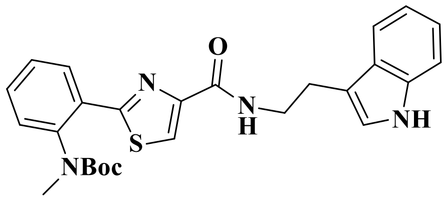
O
N
S
N
H
NH
NBoc

(6d)

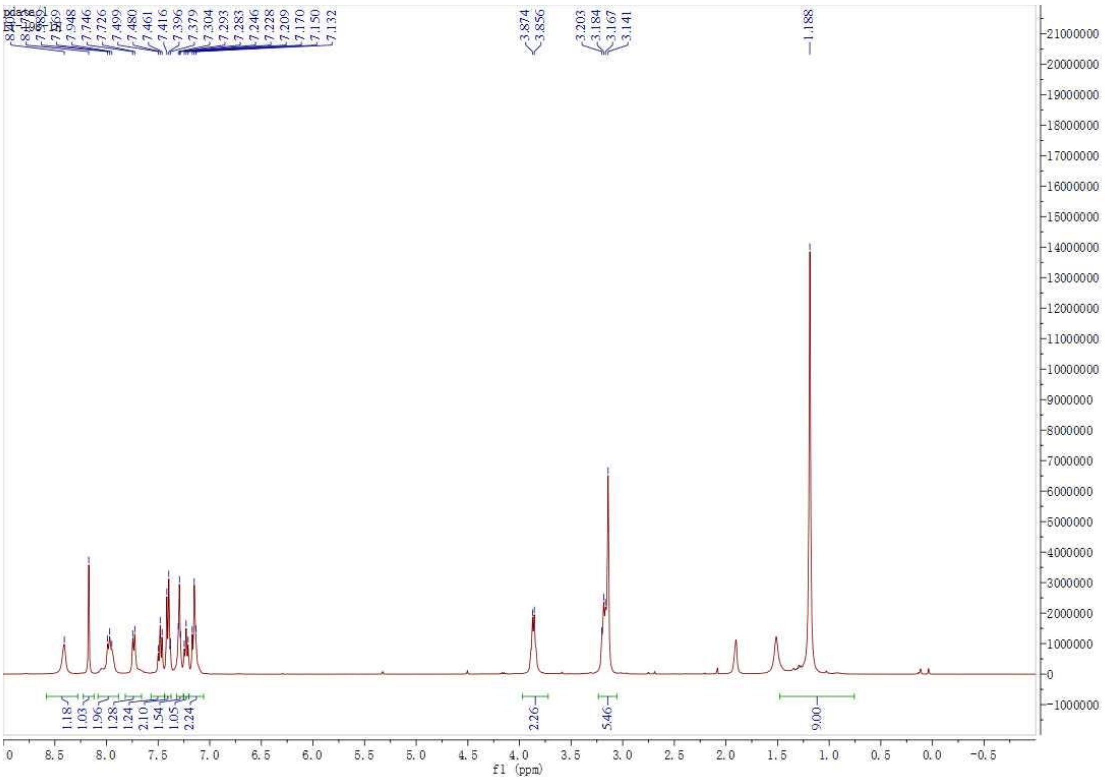
7.948
7.746
7.726
7.499
7.480
7.461
7.416
7.396
7.379
7.304
7.293
7.283
7.246
7.228
7.209
7.170
7.150
7.132
3.874
3.856
3.203
3.184
3.167
3.141
1.188
1.18
1.03
1.96
1.28
1.24
2.10
1.54
1.05
2.24
2.26
5.46
9.00
f1 (ppm)

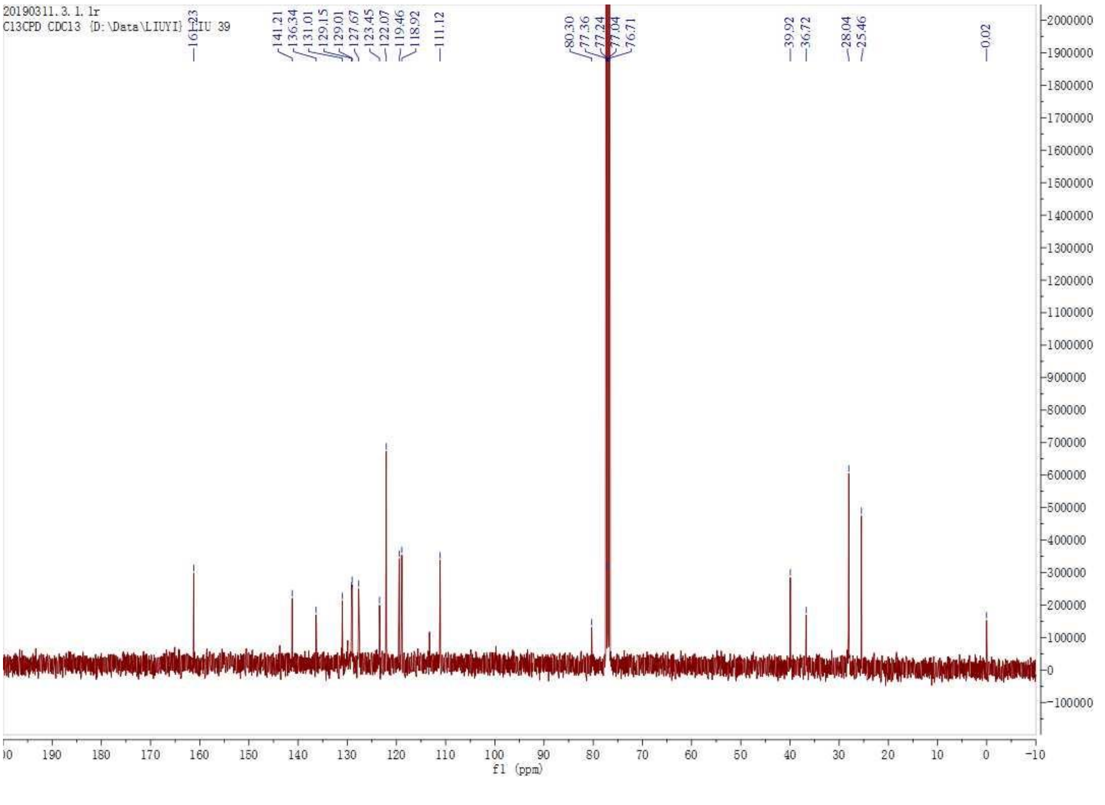
20190311.3.1.1r
C13CPD CDCl3 {D:\Data\LIUYI} LIU 39
161.23
141.21
136.34
131.01
129.15
129.01
127.67
123.45
122.07
119.46
118.92
111.12
80.30
77.36
77.24
77.04
76.71
39.92
36.72
28.04
25.46
0.02
f1 (ppm)

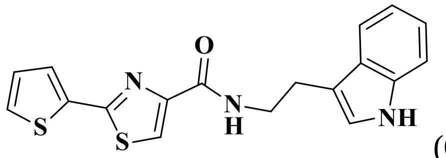
O
N
N
H
S
S
NH

(6e)

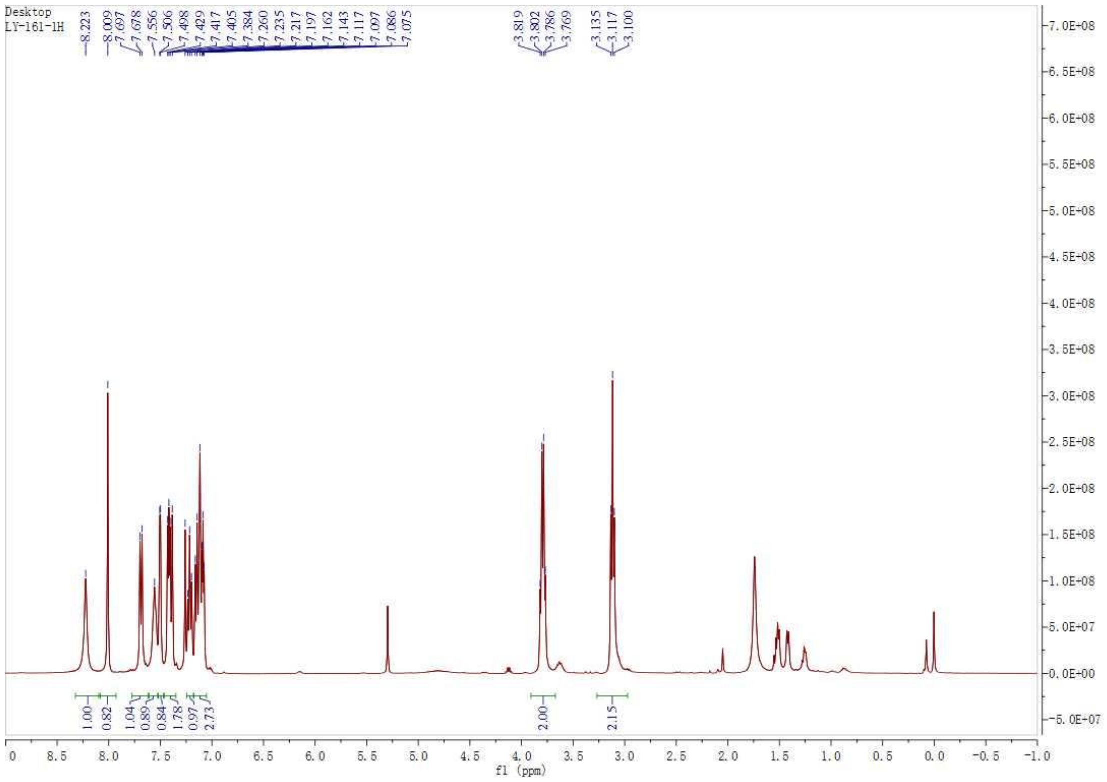
Desktop
LY-161-1H
8.223
8.009
7.697
7.678
7.556
7.506
7.498
7.429
7.417
7.405
7.384
7.260
7.235
7.217
7.197
7.162
7.143
7.117
7.097
7.086
7.075
3.819
3.802
3.786
3.769
3.135
3.117
3.100
1.00
0.82
1.04
0.89
0.84
1.78
0.97
2.73
2.00
2.15
f1 (ppm)

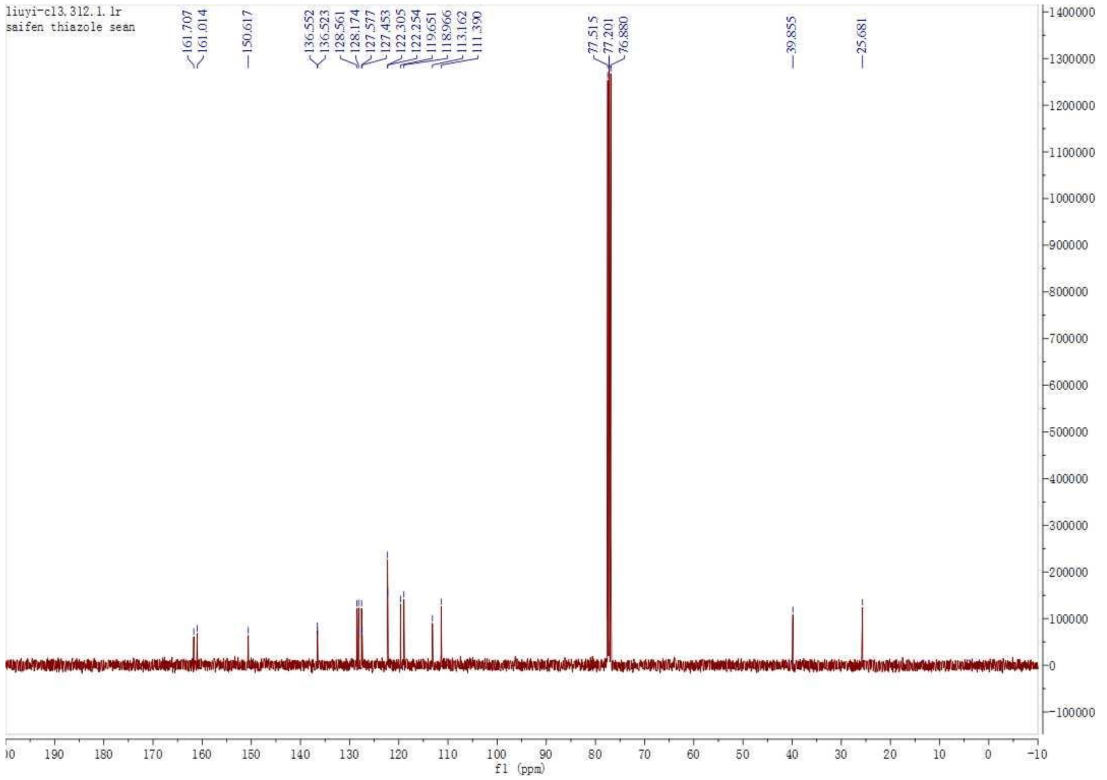
liuyi-c13.312.1.1r
saifen thiazole sean
161.707
161.014
150.617
136.552
136.523
128.561
128.174
127.577
127.453
122.305
122.254
119.651
118.966
113.162
111.390
77.515
77.201
76.880
39.855
25.681
f1 (ppm)

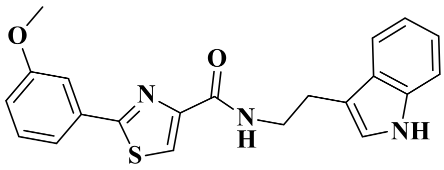
O
O
N
N
H
S
NH

(6f)

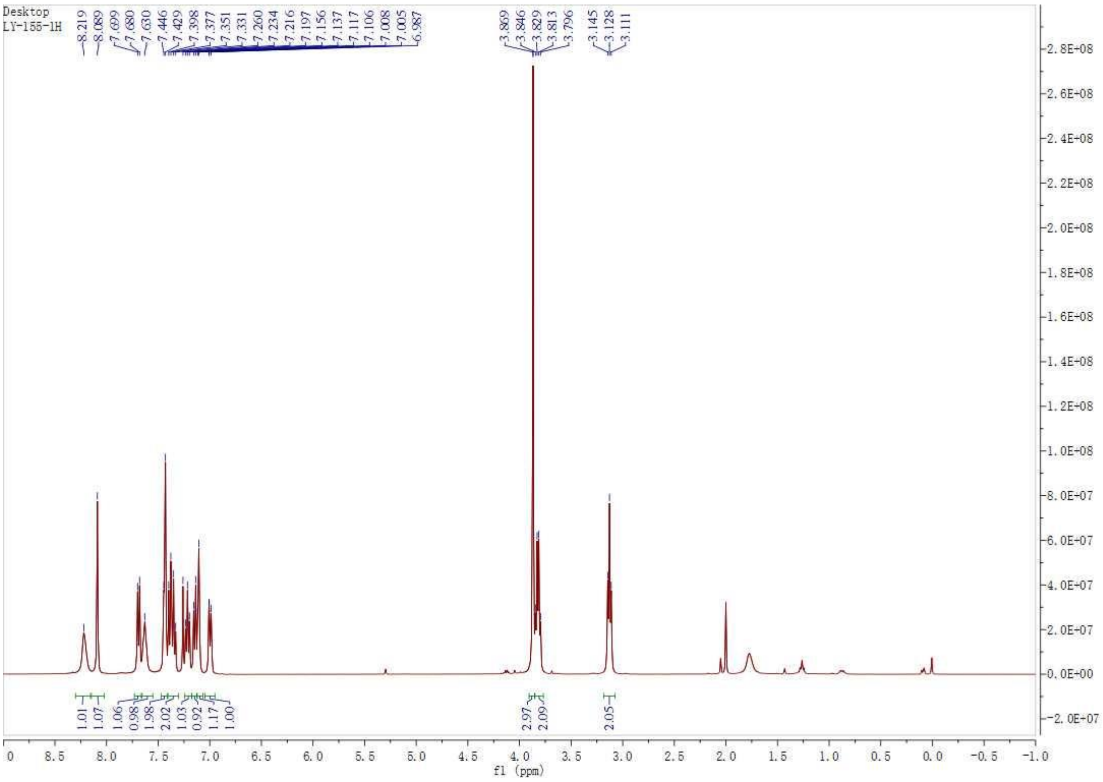
Desktop
LY-155-1H
8.219 8.089 7.699 7.680 7.630 7.446 7.429 7.398 7.377 7.351 7.331 7.260 7.234 7.216 7.197 7.156 7.137 7.117 7.106 7.008 7.005 6.987
3.869 3.846 3.829 3.813 3.796
3.145 3.128 3.111
1.01 1.07 1.06 0.98 1.98 2.02 1.03 0.92 1.17 1.00
2.97 2.09
2.05
f1 (ppm)

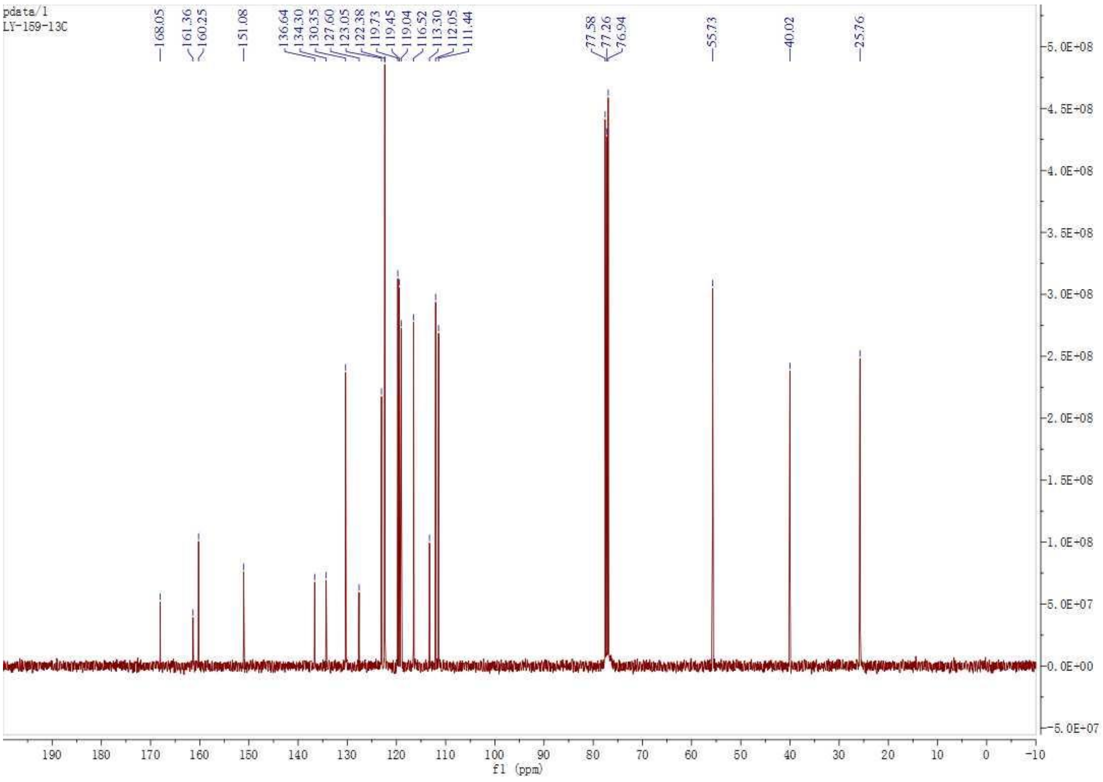
pdata/1
LY-159-13C
168.05
161.36 160.25
151.08
136.64 134.30 130.35 127.60 123.05 122.38 119.73 119.45 119.04 116.52 113.30 112.05 111.44
77.58 77.26 76.94
55.73
40.02
25.76
f1 (ppm)

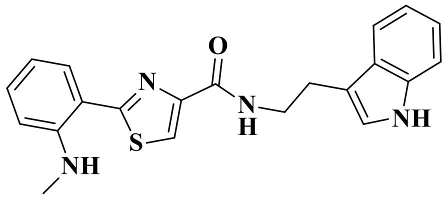

(6g)

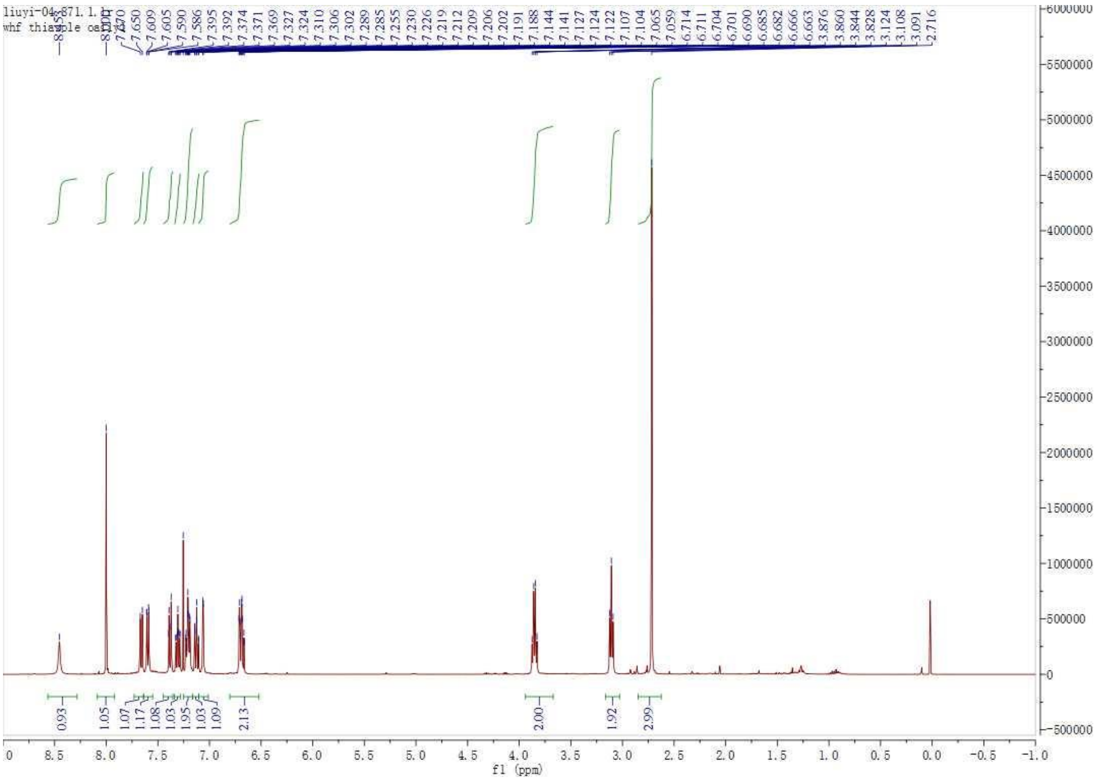

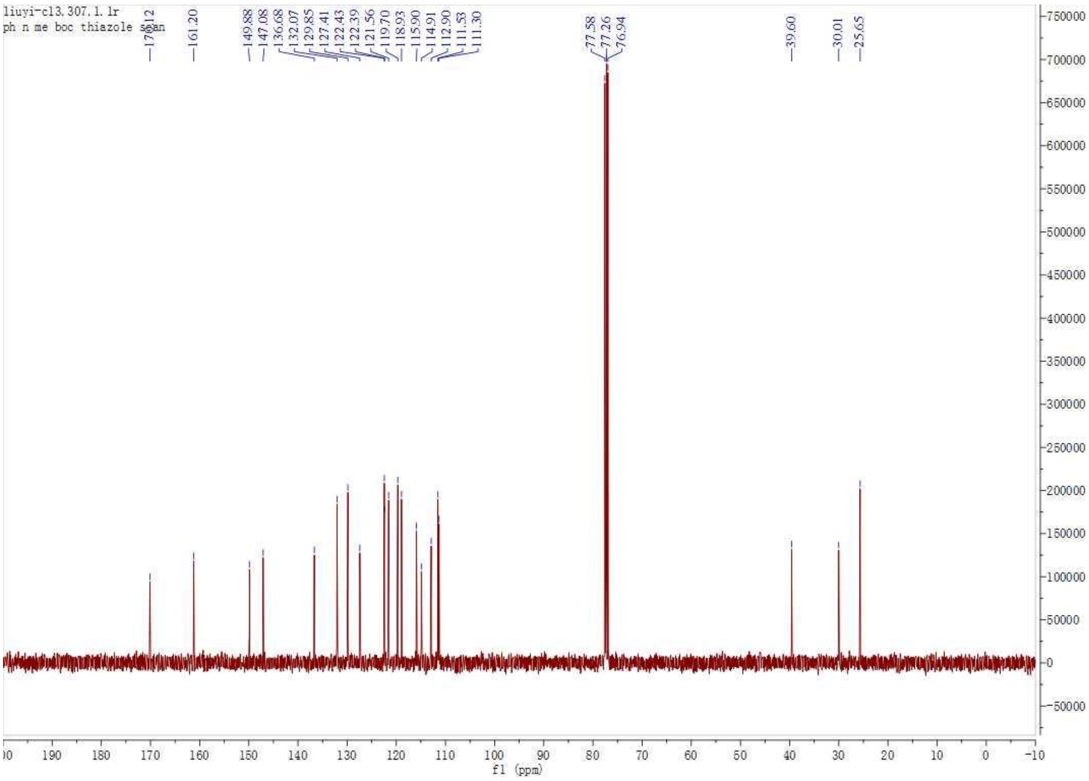

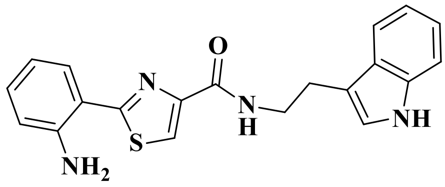
O
N
N
H
NH
S
NH2

(6h)

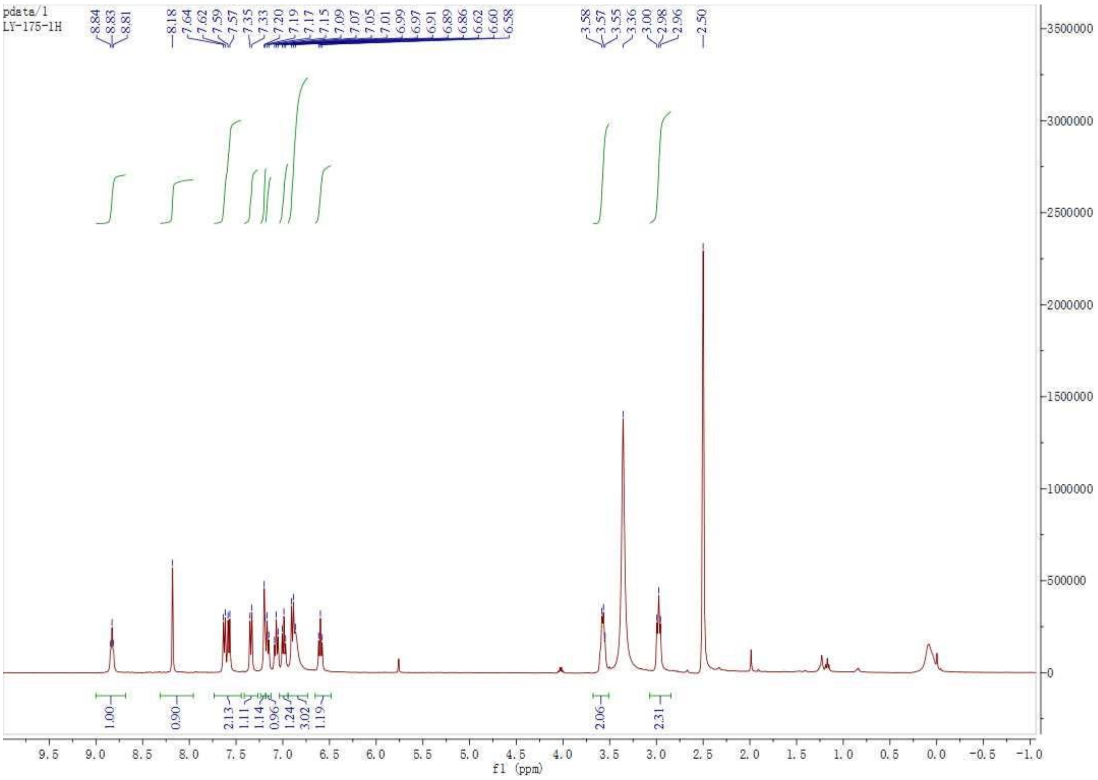
pdata/1
LY-175-1H
8.84 8.83 8.81 8.18 7.64 7.62 7.59 7.57 7.35 7.33 7.20 7.19 7.17 7.15 7.09 7.07 7.05 7.01 6.99 6.97 6.91 6.89 6.86 6.62 6.60 6.58
3.58 3.57 3.55 3.36 3.00 2.98 2.96 2.50
1.00 0.90 2.13 1.11 1.14 0.96 1.24 3.02 1.19 2.06 2.31
f1 (ppm)

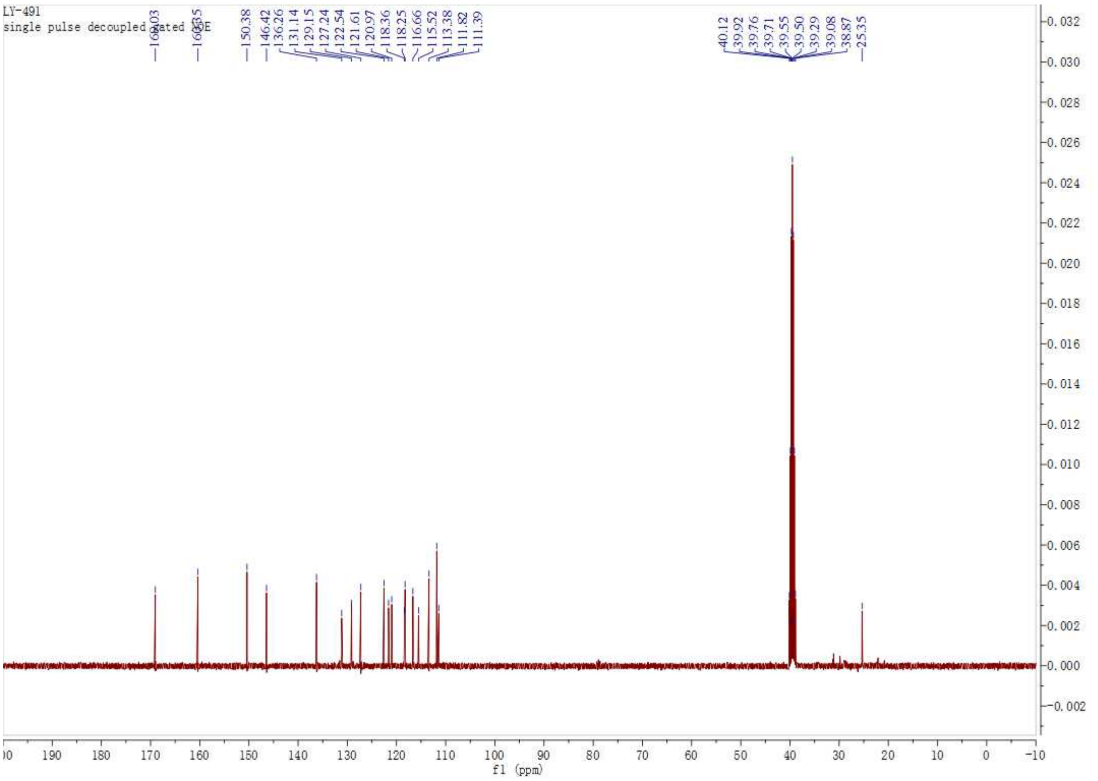
LY-491
single pulse decoupled gated NOE
169.03 160.35 150.38 146.42 136.26 131.14 129.15 127.24 122.54 121.61 120.97 118.36 118.25 116.66 115.52 113.38 111.82 111.39
40.12 39.92 39.76 39.71 39.55 39.50 39.29 39.08 38.87 25.35
f1 (ppm)

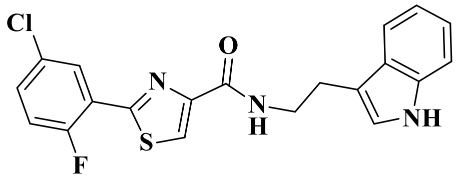
Cl
O
N
N
H
S
F
NH

(6i)

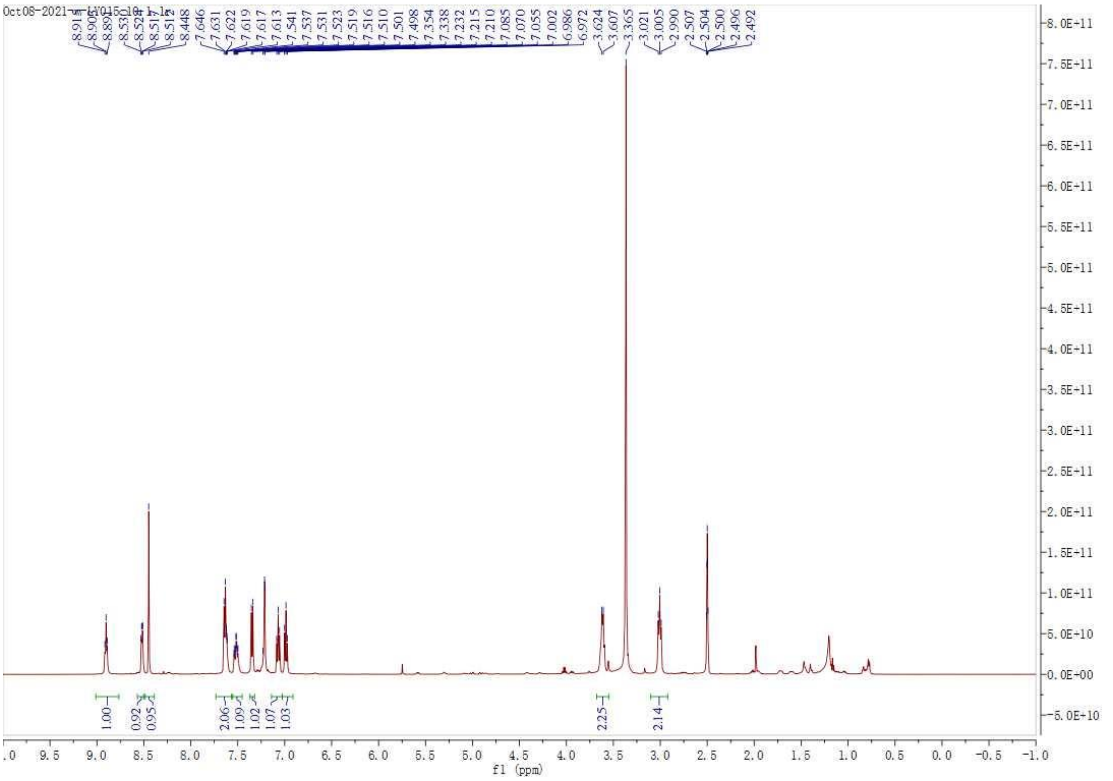
Oct08-2021-w-LY015.10.1.1r
8.915
8.900
8.889
8.531
8.521
8.517
8.512
8.448
7.646
7.631
7.622
7.619
7.617
7.613
7.541
7.537
7.531
7.523
7.519
7.516
7.510
7.501
7.498
7.354
7.338
7.232
7.215
7.210
7.085
7.070
7.055
7.002
6.986
6.972
3.624
3.607
3.365
3.021
3.005
2.990
2.507
2.504
2.500
2.496
2.492
1.00
0.92
0.95
2.06
1.09
1.02
1.07
1.03
2.25
2.14
f1 (ppm)

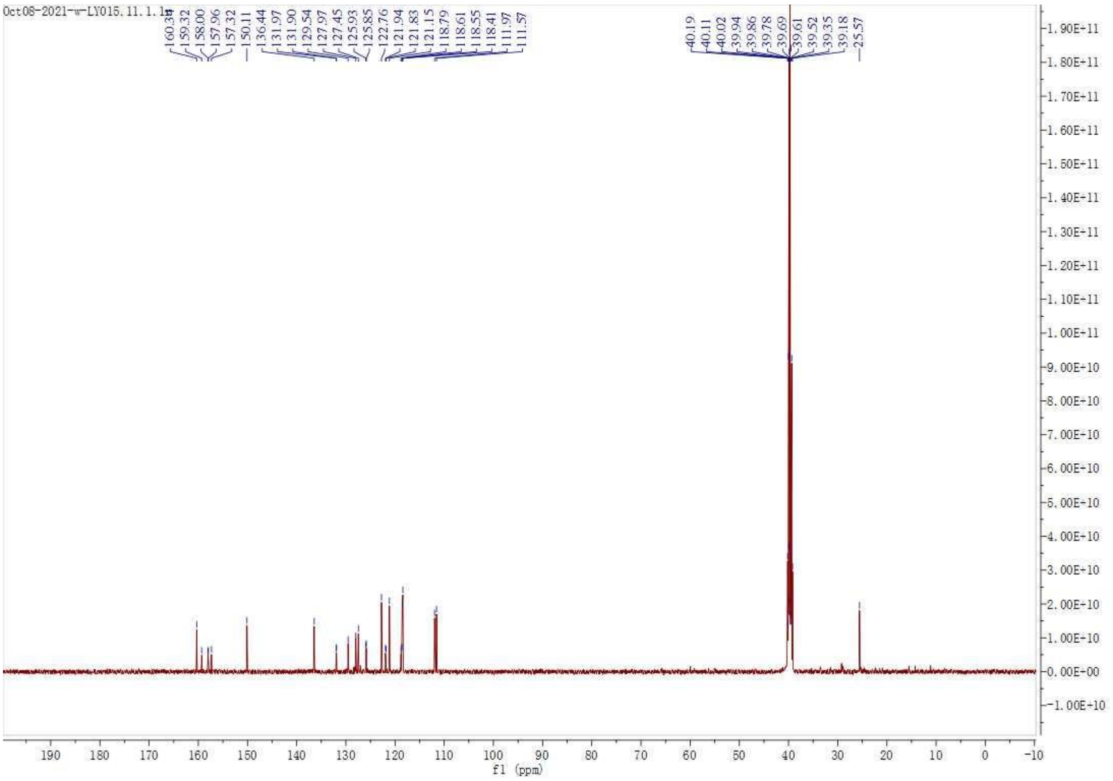
Oct08-2021-w-LY015.11.1.1r
160.34
159.32
158.00
157.96
157.32
150.11
136.44
131.97
131.90
129.54
127.97
127.45
125.93
125.85
122.76
121.94
121.83
121.15
118.79
118.61
118.55
118.41
111.97
111.57
40.19
40.11
40.02
39.94
39.86
39.78
39.69
39.61
39.52
39.35
39.18
25.57
f1 (ppm)

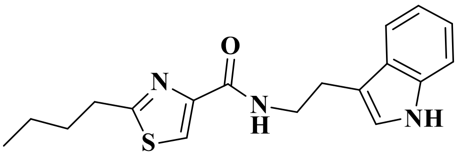

(6j)

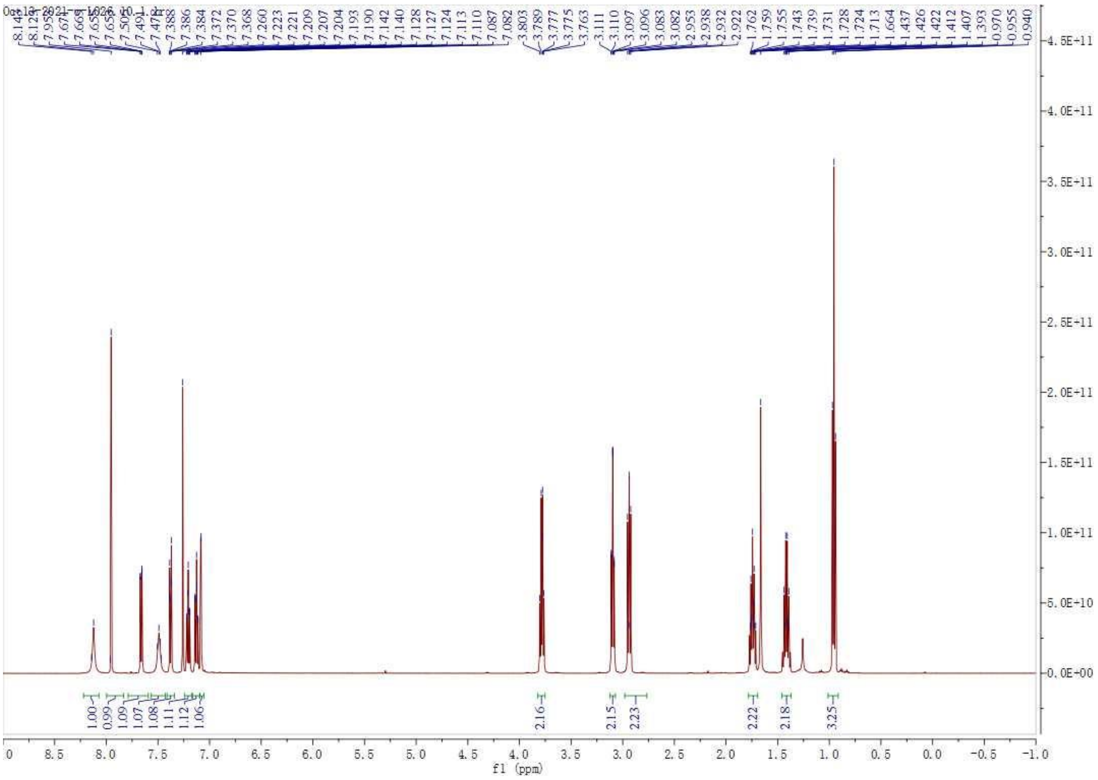

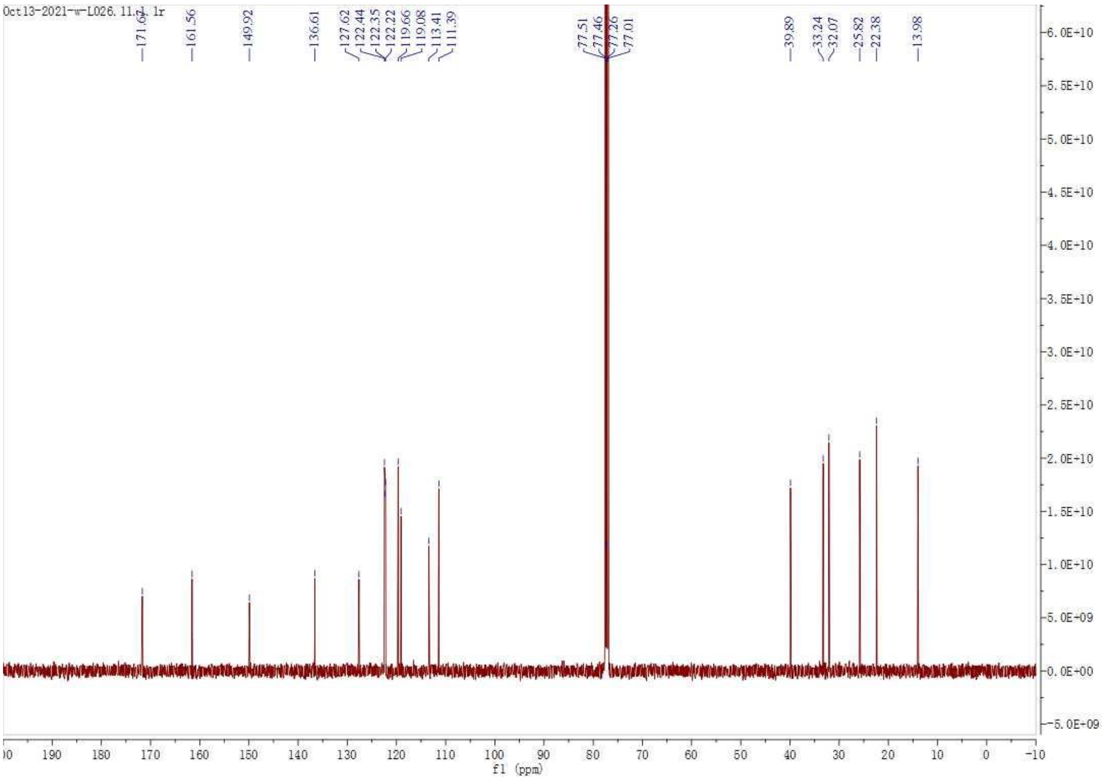

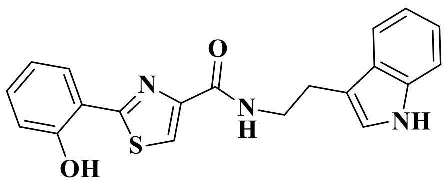

(6k)

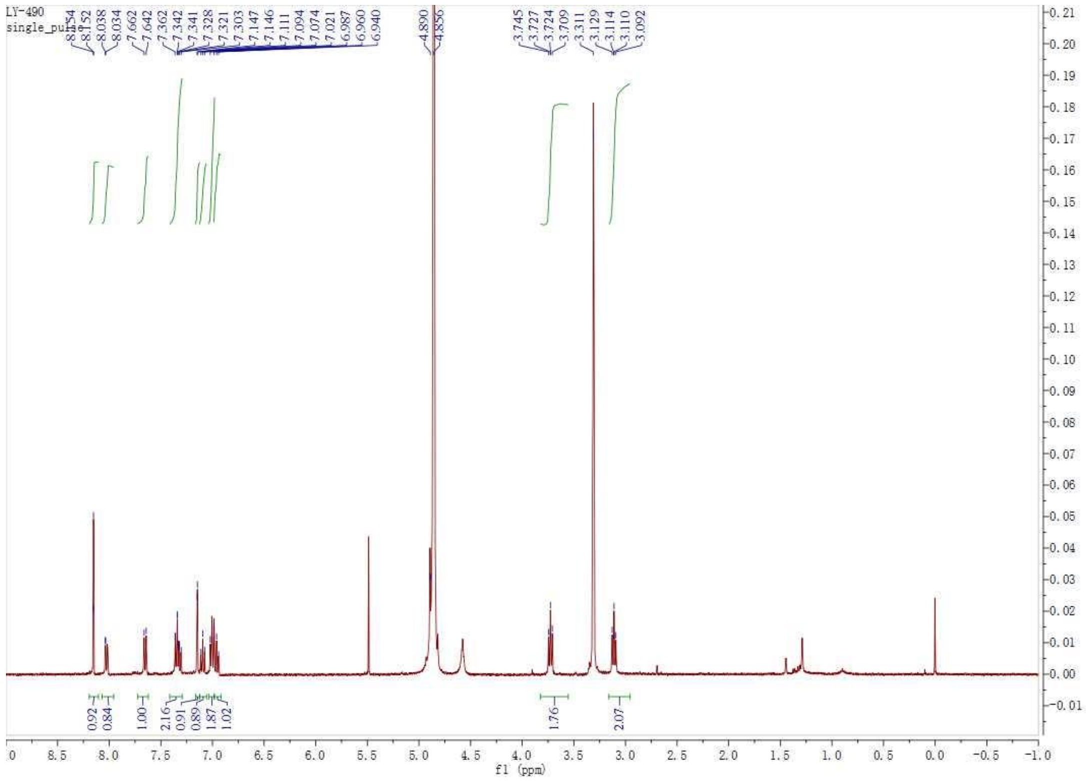

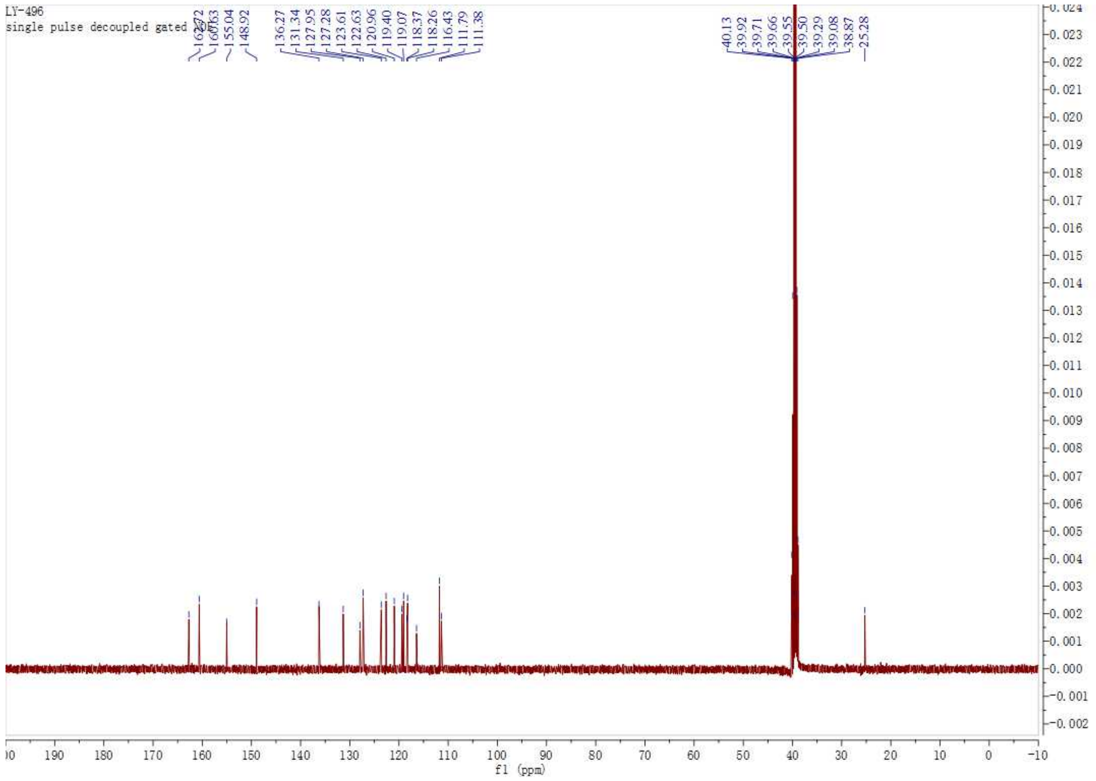

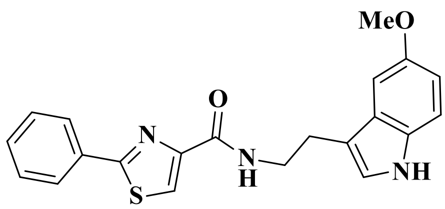
MeO
O
N
S
N
H
NH

(7b)

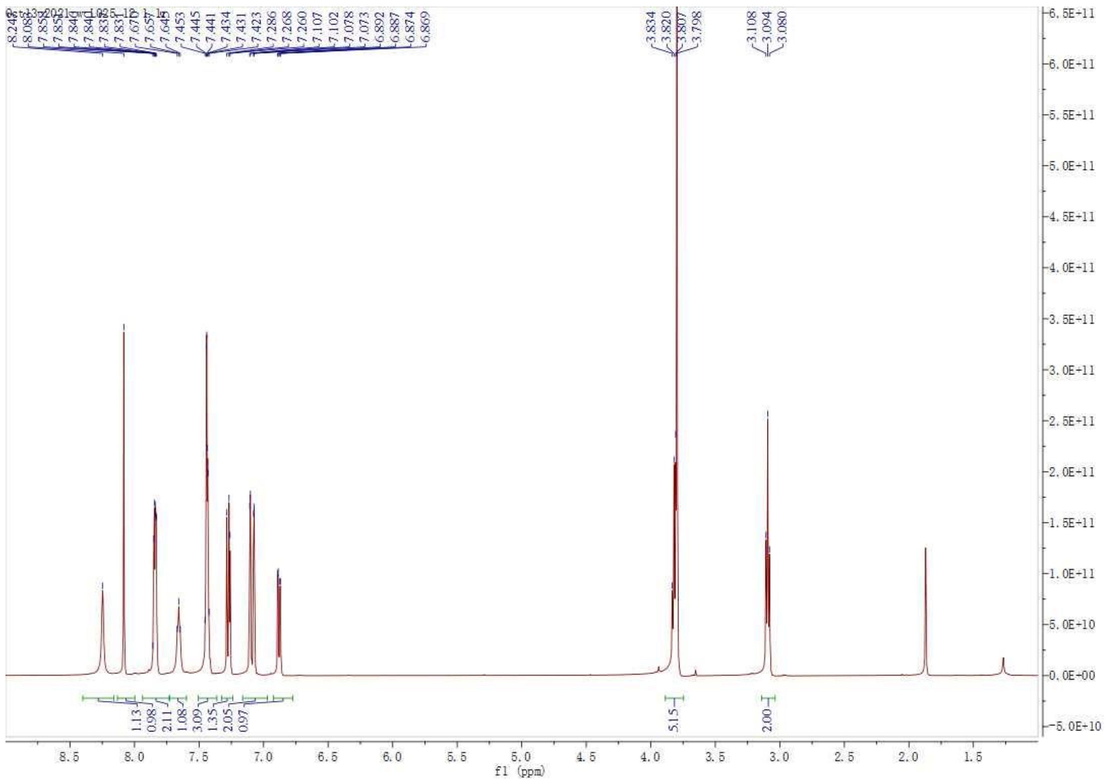
3.834
3.820
3.807
3.798
3.108
3.094
3.080
1.13
0.98
2.11
1.08
3.09
1.35
2.05
0.97
5.15
2.00
f1 (ppm)

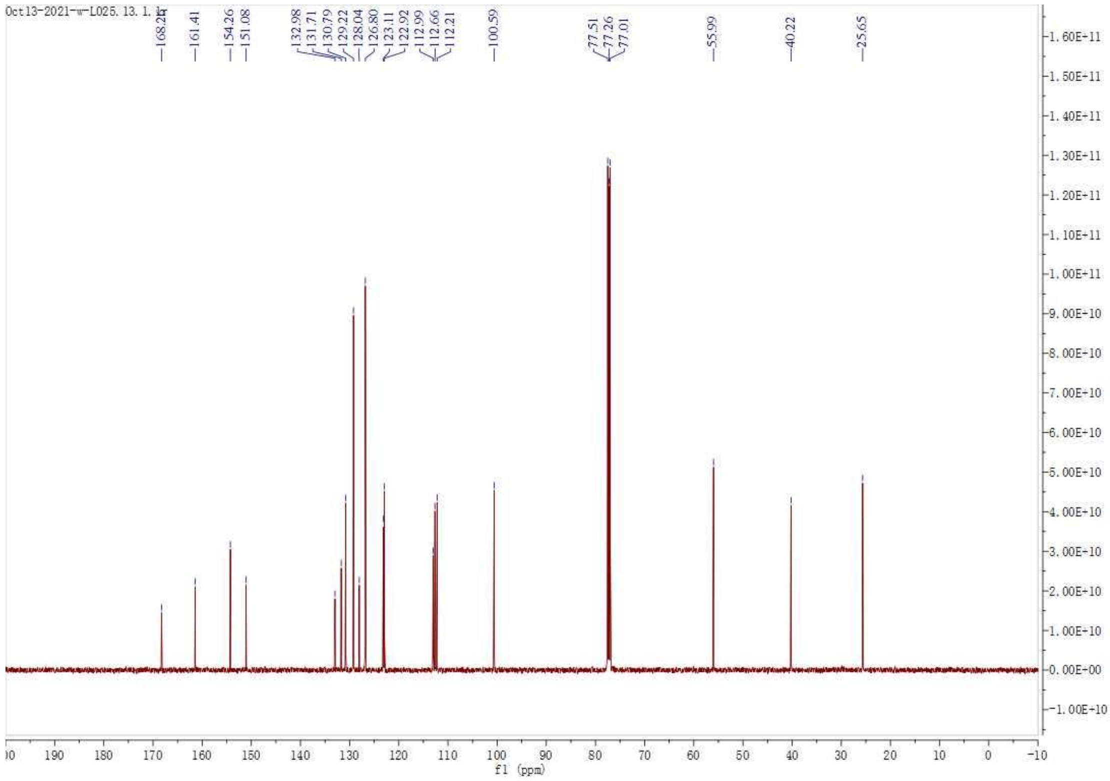
168.24
161.41
154.26
151.08
132.98
131.71
130.79
129.22
128.04
126.80
123.11
122.92
112.99
112.66
112.21
100.59
77.51
77.26
77.01
55.99
40.22
25.65
f1 (ppm)

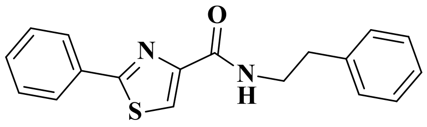

(7c)

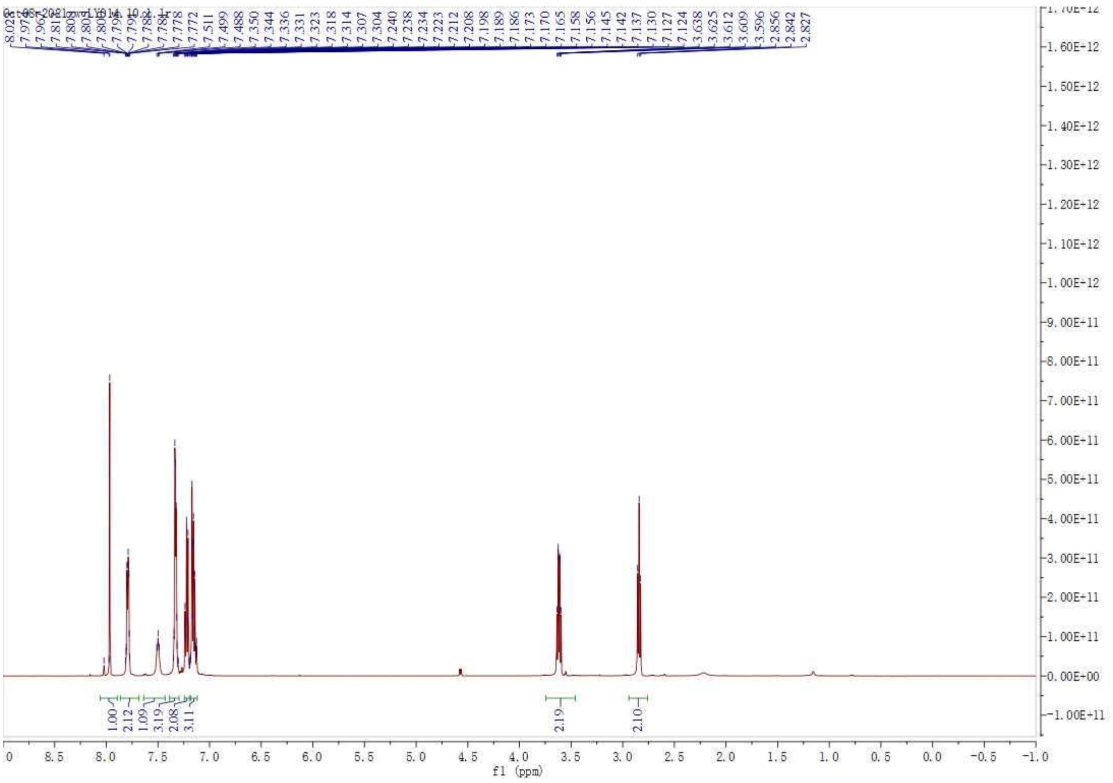

1.00
2.12
1.09
3.19
2.08
3.11
2.19
2.10
f1 (ppm)

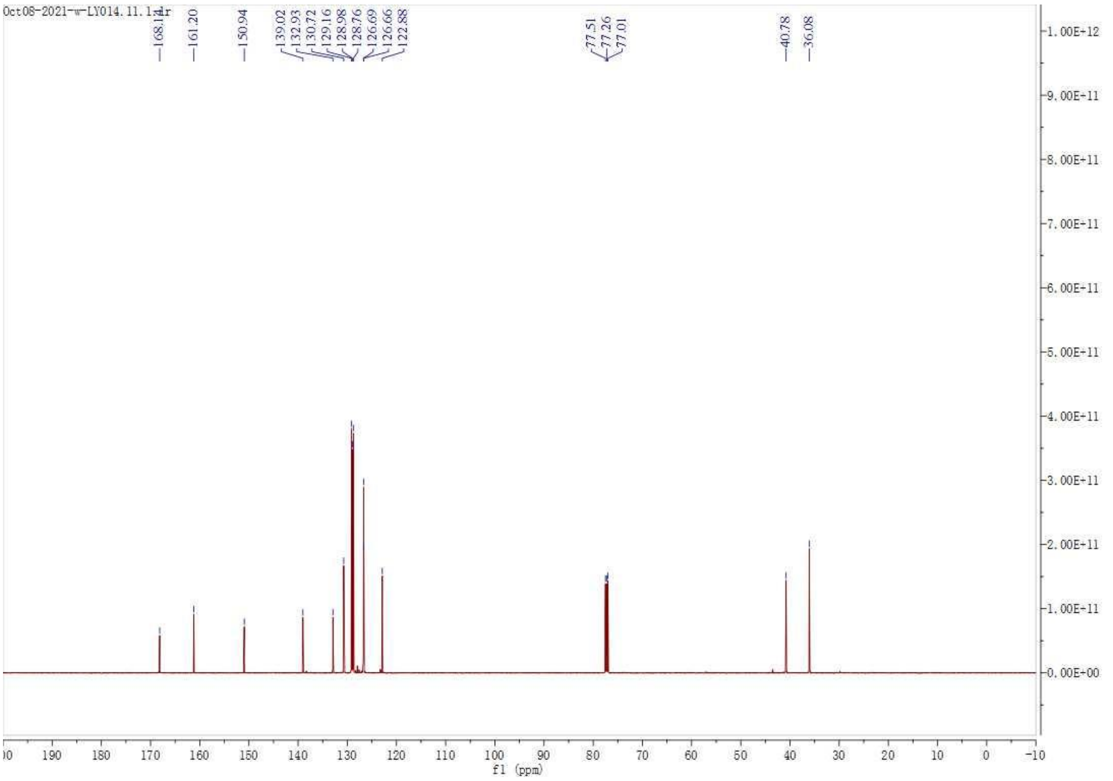

Oct08-2021-w-LY014.11.1.fid
168.11
161.20
150.94
139.02
132.93
130.72
129.16
128.98
128.76
126.69
126.66
122.88
77.51
77.26
77.01
40.78
36.08
f1 (ppm)

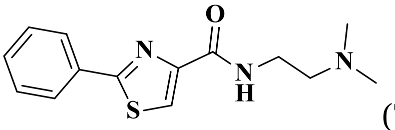
O
N
N
H
S
N

(7d)

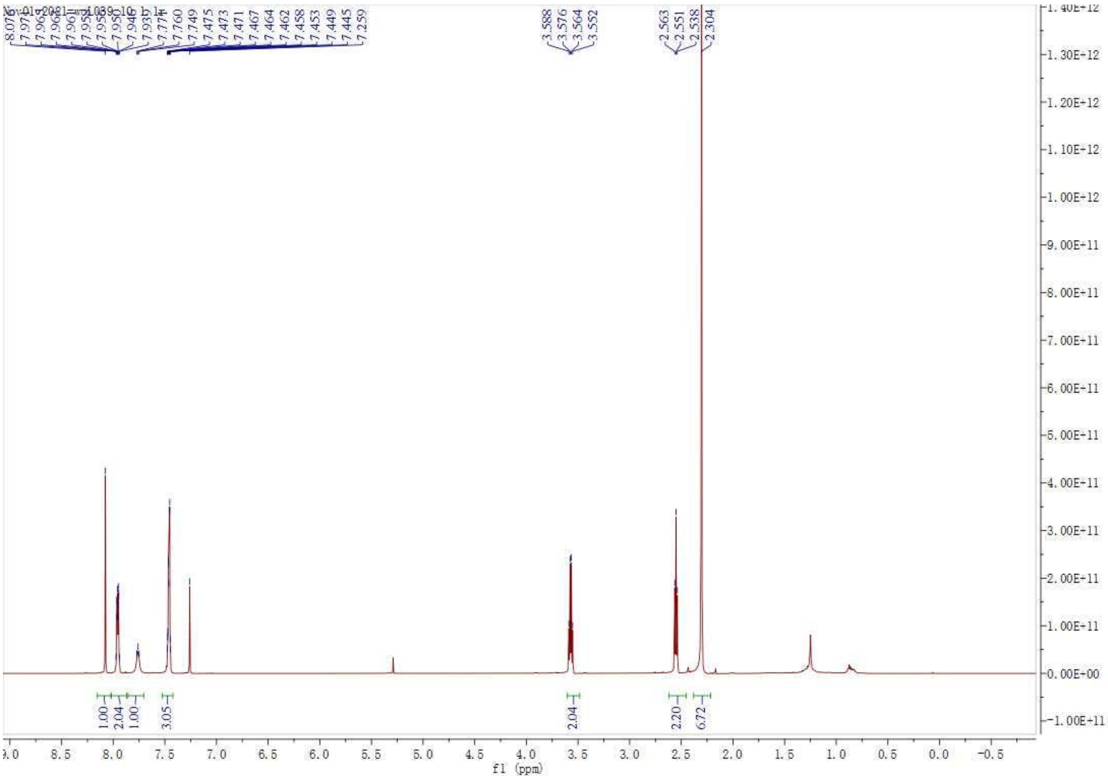
3.588
3.576
3.564
3.552
2.563
2.551
2.538
2.304
1.00
2.04
1.00
3.05
2.04
2.20
6.72
f1 (ppm)

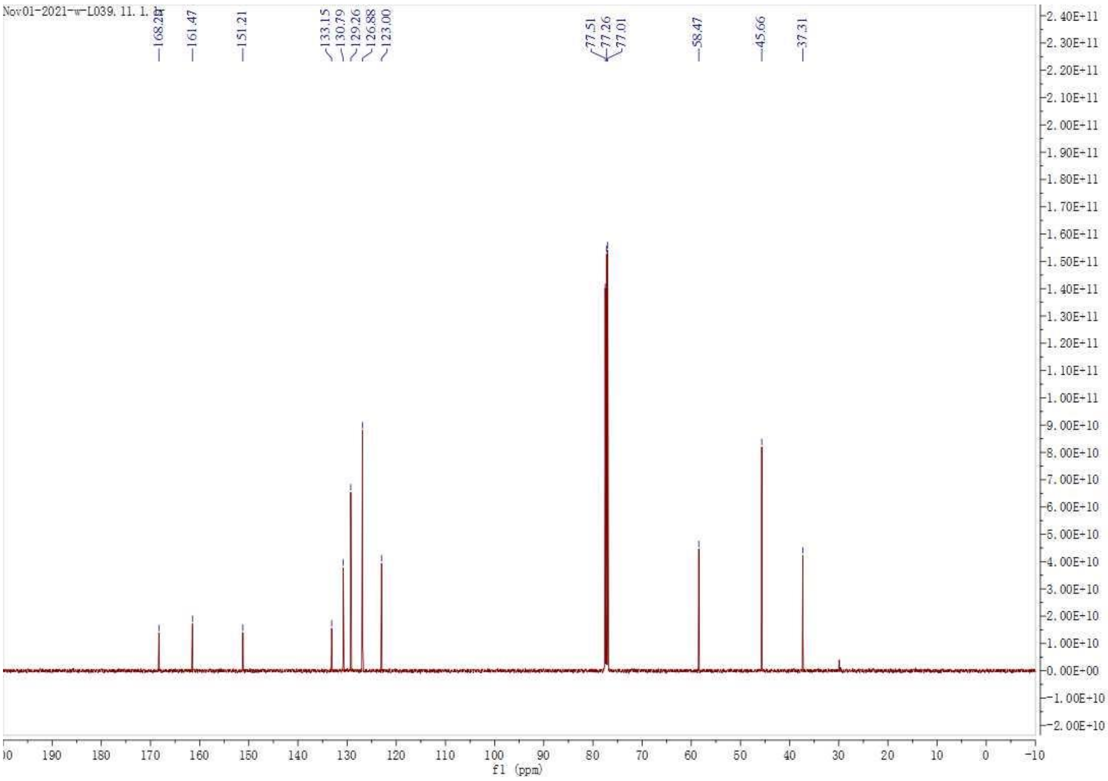
Nov01-2021-w-L039.11.1.1r
161.47
151.21
133.15
130.79
129.26
126.88
123.00
77.51
77.26
77.01
58.47
45.66
37.31
f1 (ppm)

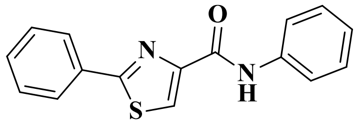
O
N
S
N
H

(7e)

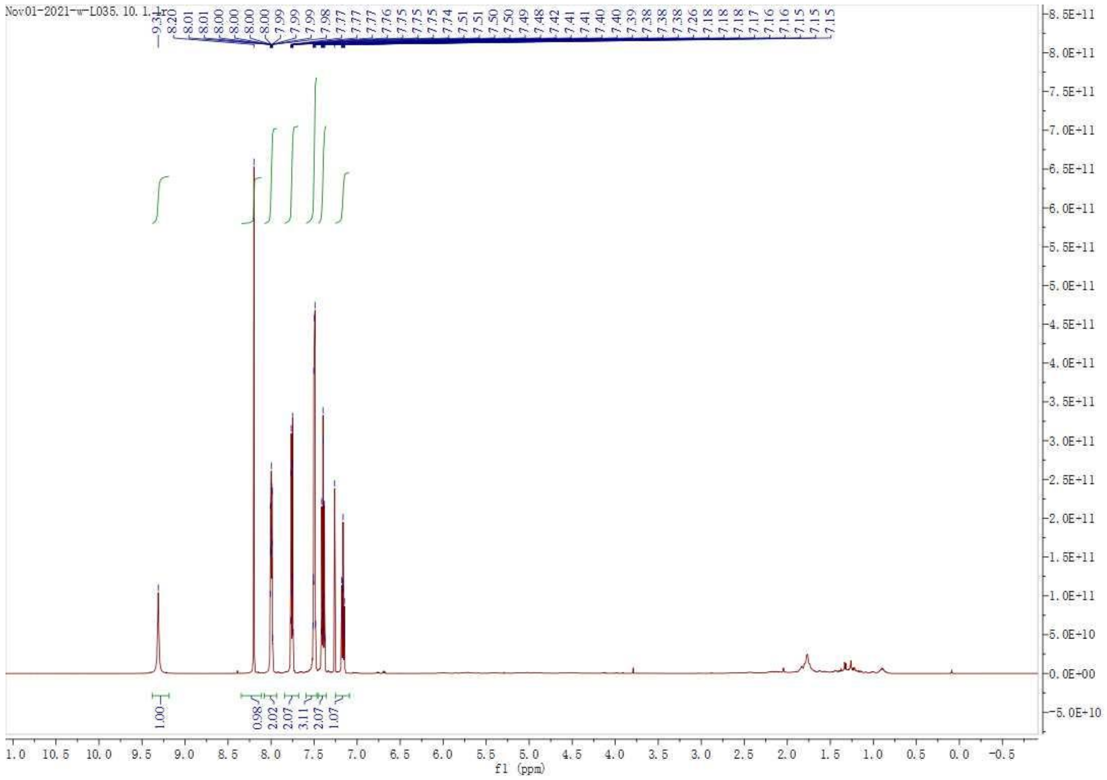
Nov01-2021-w-L035.10.1.1r
9.31
8.20
8.01
8.01
8.00
8.00
8.00
7.99
7.99
7.98
7.77
7.77
7.76
7.75
7.75
7.74
7.51
7.51
7.50
7.50
7.49
7.48
7.42
7.41
7.40
7.40
7.39
7.38
7.38
7.38
7.26
7.18
7.18
7.18
7.17
7.16
7.15
7.15
7.15
1.00
0.98
2.02
2.07
3.11
2.07
1.07
f1 (ppm)

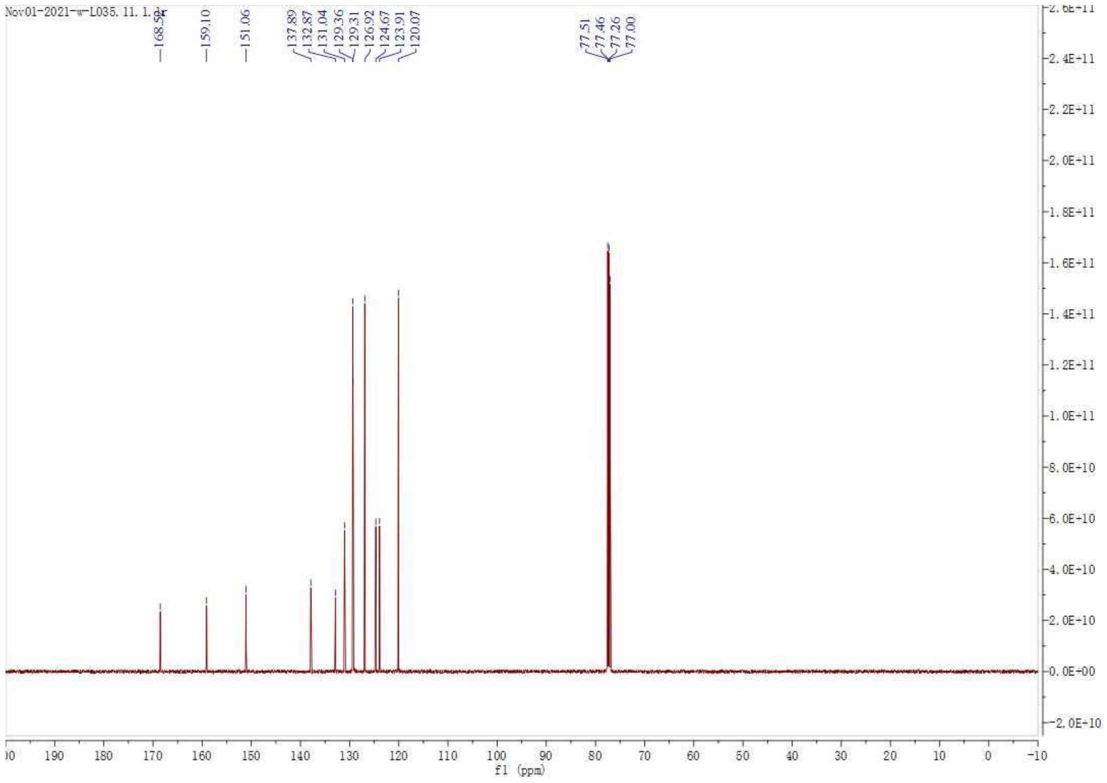
Nov01-2021-w-L035.11.1.1r
168.52
159.10
151.06
137.89
132.87
131.04
129.36
129.31
126.92
124.67
123.91
120.07
77.51
77.46
77.26
77.00
f1 (ppm)

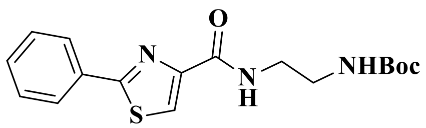

(7f)

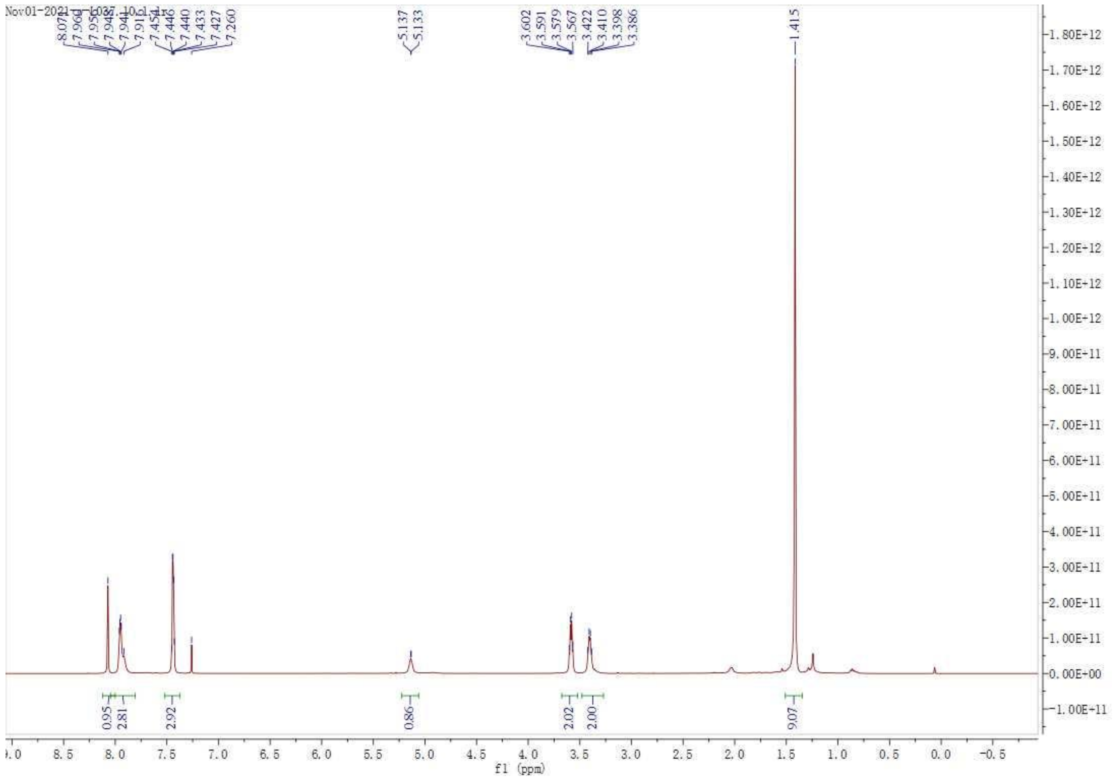

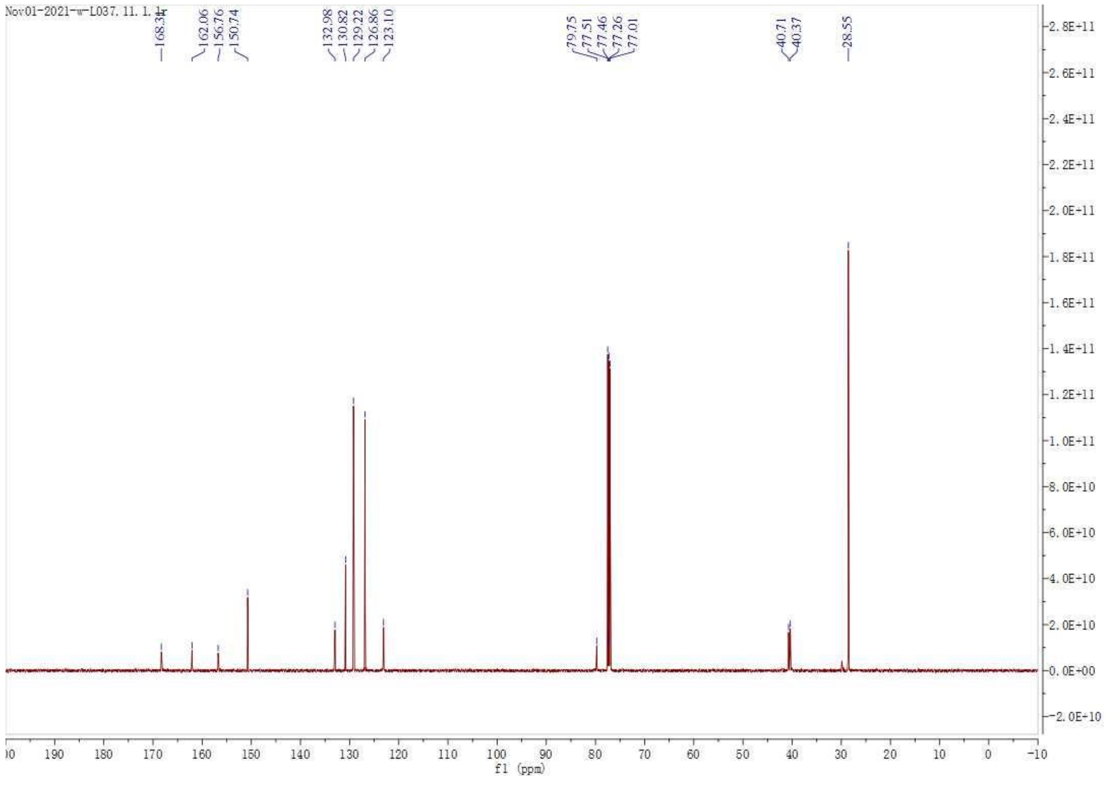

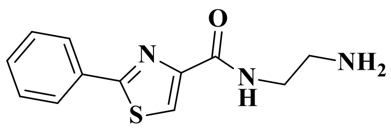

(7g)

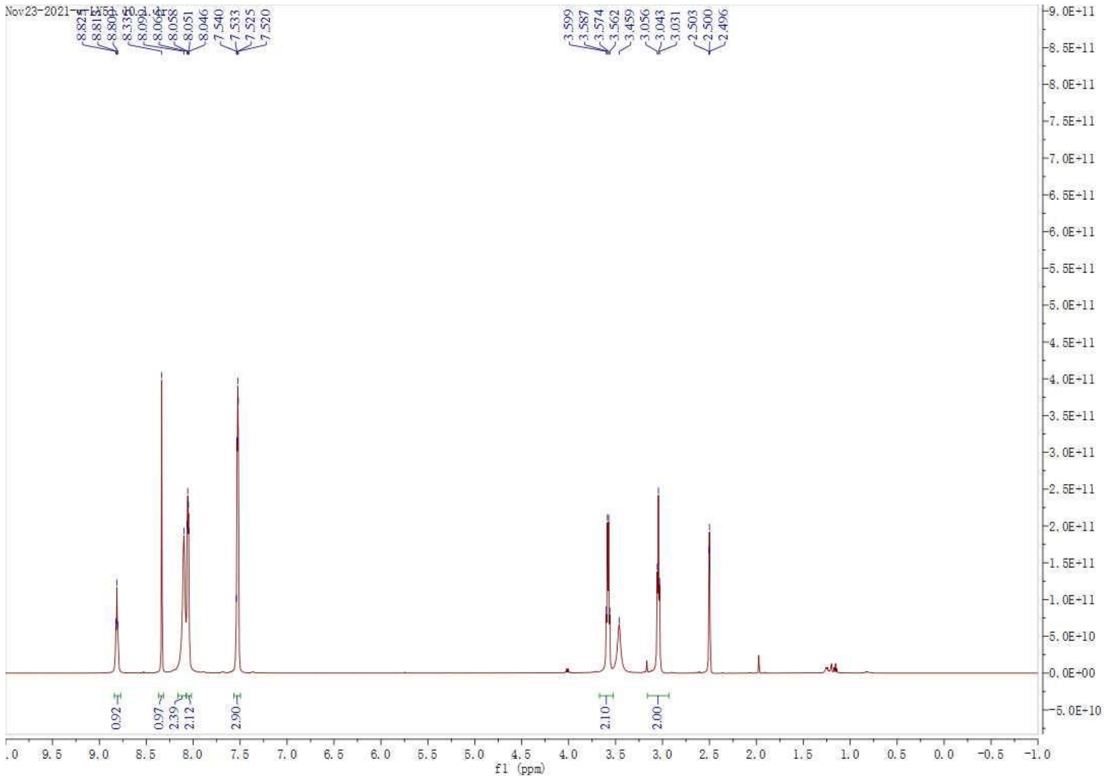

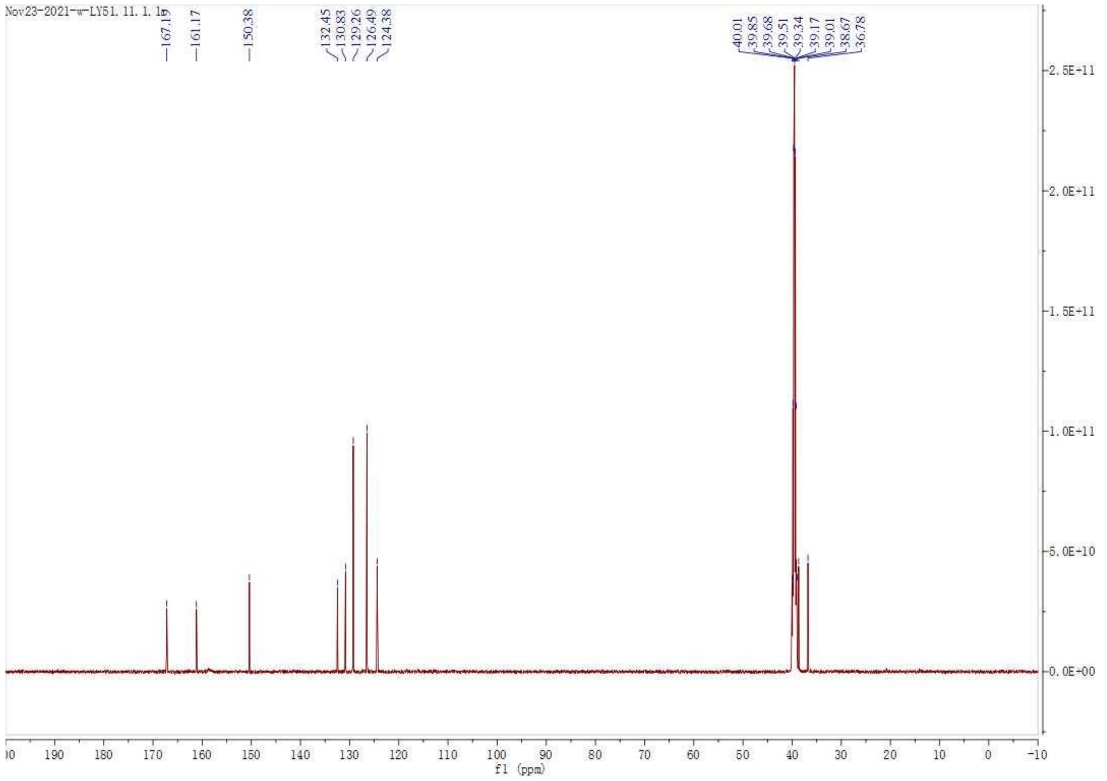

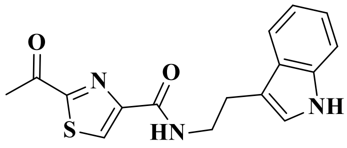

(Bacillamide A)

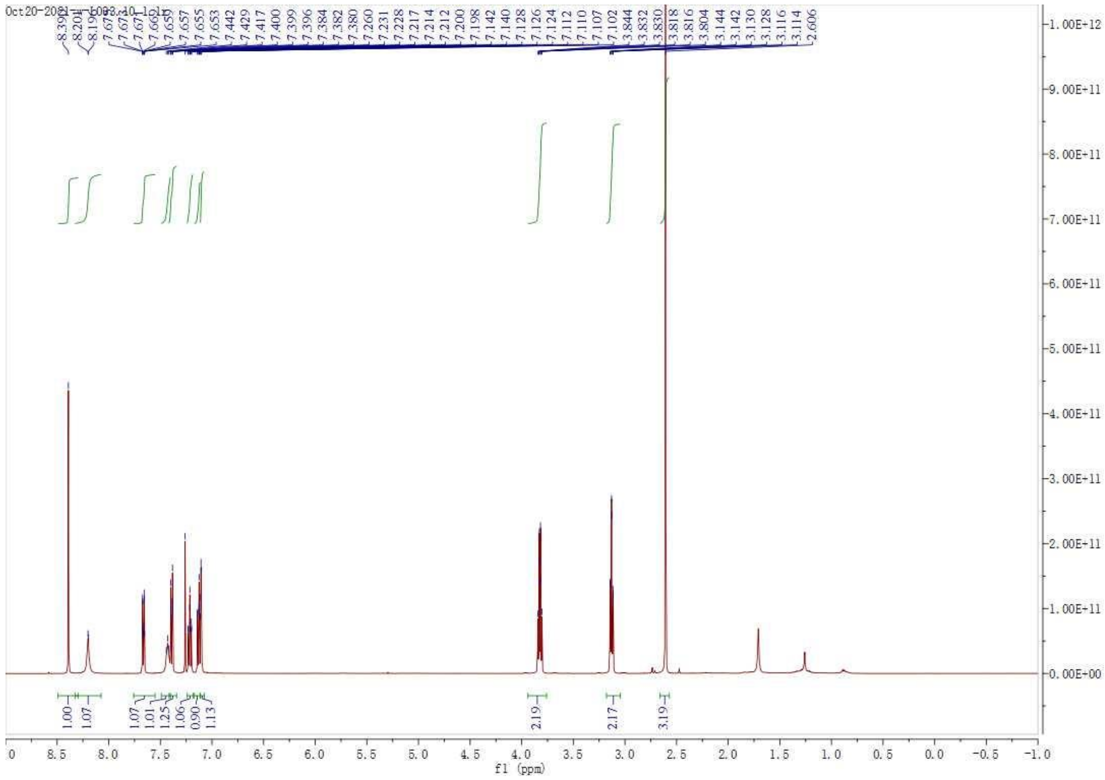

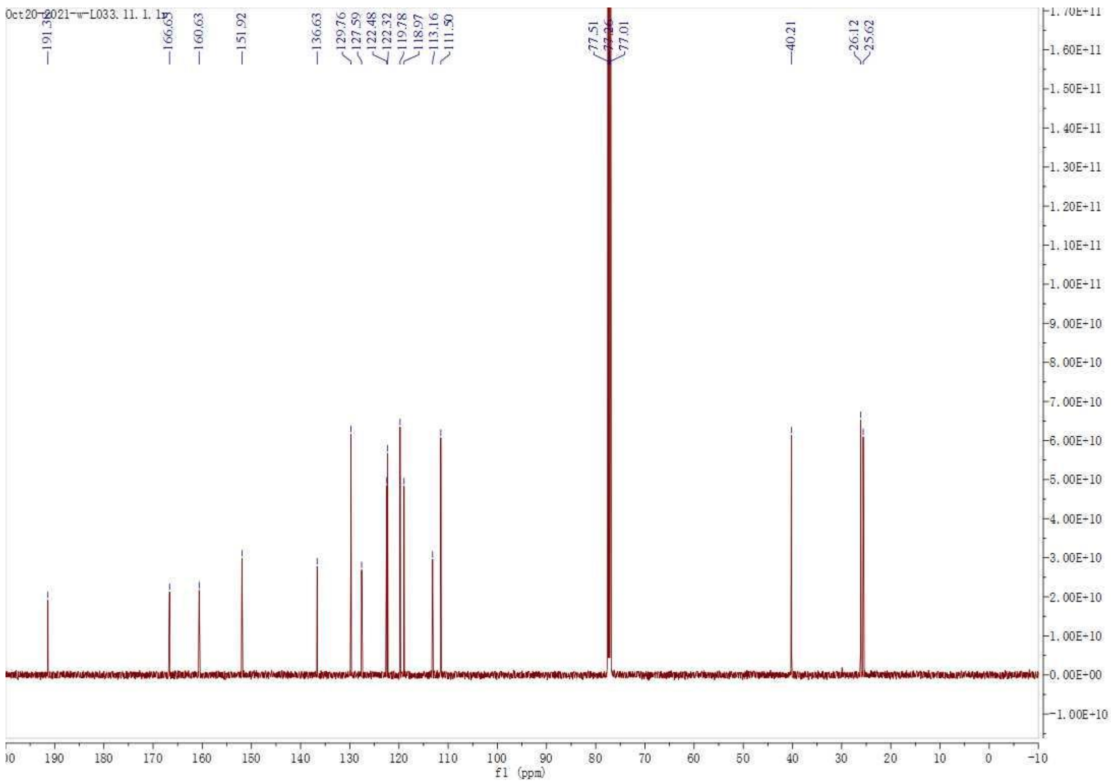

Supplement: Supplementary file 1 [file marinedrugs-22-00494-s001.zip › marinedrugs-3280917-supplementary.pdf]
